# A Neuroimaging Web Services Interface as a Cyber Physical System for Medical Imaging and Data Management in Brain Research: Design Study

**DOI:** 10.2196/medinform.9063

**Published:** 2018-04-26

**Authors:** Gabriel Lizarraga, Chunfei Li, Mercedes Cabrerizo, Warren Barker, David A Loewenstein, Ranjan Duara, Malek Adjouadi

**Affiliations:** ^1^ Center for Advanced Technology and Education Computer Science Florida International University Miami, FL United States; ^2^ Wien Center for Alzheimer's Disease & Memory Disorders Mount Sinai Medical Center Miami Beach, FL United States; ^3^ Center on Aging and Department of Psychiatry and Behavioral Sciences Miller School of Medicine University of Miami Miami, FL United States

**Keywords:** neuroimaging, multimodal imaging, magnetic resonance imaging, image processing, positron-emission tomography, diffusion tensor imaging, information storage and retrieval, diagnostic imaging

## Abstract

**Background:**

Structural and functional brain images are essential imaging modalities for medical experts to study brain anatomy. These images are typically visually inspected by experts. To analyze images without any bias, they must be first converted to numeric values. Many software packages are available to process the images, but they are complex and difficult to use. The software packages are also hardware intensive. The results obtained after processing vary depending on the native operating system used and its associated software libraries; data processed in one system cannot typically be combined with data on another system.

**Objective:**

The aim of this study was to fulfill the neuroimaging community’s need for a common platform to store, process, explore, and visualize their neuroimaging data and results using Neuroimaging Web Services Interface: a series of processing pipelines designed as a cyber physical system for neuroimaging and clinical data in brain research.

**Methods:**

Neuroimaging Web Services Interface accepts magnetic resonance imaging, positron emission tomography, diffusion tensor imaging, and functional magnetic resonance imaging. These images are processed using existing and custom software packages. The output is then stored as image files, tabulated files, and MySQL tables. The system, made up of a series of interconnected servers, is password-protected and is securely accessible through a Web interface and allows (1) visualization of results and (2) downloading of tabulated data.

**Results:**

All results were obtained using our processing servers in order to maintain data validity and consistency. The design is responsive and scalable. The processing pipeline started from a FreeSurfer reconstruction of Structural magnetic resonance imaging images. The FreeSurfer and regional standardized uptake value ratio calculations were validated using Alzheimer’s Disease Neuroimaging Initiative input images, and the results were posted at the Laboratory of Neuro Imaging data archive. Notable leading researchers in the field of Alzheimer’s Disease and epilepsy have used the interface to access and process the data and visualize the results. Tabulated results with unique visualization mechanisms help guide more informed diagnosis and expert rating, providing a truly unique multimodal imaging platform that combines magnetic resonance imaging, positron emission tomography, diffusion tensor imaging, and resting state functional magnetic resonance imaging. A quality control component was reinforced through expert visual rating involving at least 2 experts.

**Conclusions:**

To our knowledge, there is no validated Web-based system offering all the services that Neuroimaging Web Services Interface offers. The intent of Neuroimaging Web Services Interface is to create a tool for clinicians and researchers with keen interest on multimodal neuroimaging. More importantly, Neuroimaging Web Services Interface significantly augments the Alzheimer’s Disease Neuroimaging Initiative data, especially since our data contain a large cohort of Hispanic normal controls and Alzheimer’s Disease patients. The obtained results could be scrutinized visually or through the tabulated forms, informing researchers on subtle changes that characterize the different stages of the disease.

## Introduction

### Background

Noninvasive brain imaging modalities contribute considerably to the understanding of brain structure and functionality [1]. Magnetic resonance imaging (MRI), positron emission tomography (PET), diffusion tensor imaging (DTI), and functional magnetic resonance imaging (fMRI) scans, among other modalities, allow clinicians and experts to advance their research and take informed decisions on the diagnosis and the planning of clinical or therapeutic interventions that could follow. The images obtained by these scans must first be preprocessed in order to convert them into numeric values that can be objectively assessed and analyzed. Hospitals and other research institutions can capture, store, and view brain scans on their own picture archiving and communication system; but performing additional processing is often computationally taxing, requiring specialized software, a suitable hardware or computing infrastructure, and image processing expertise, which our Neuroimaging Web Services Interface (NWSI) is designed to offer.

In addition to the need of individual investigators to test and validate results, there is a larger neuroscience community in academia and medical settings that can benefit from this integrated processing and visualization platform. Data sharing, which remains limited due to the different institutional and privacy constraints, should be encouraged within the scientific community to increase the value of research. The governing council of the Organization for Human Brain Mapping, which is the primary international organization dedicated to neuroimaging research, highlighted in 2001 certain challenges in the field of databases in neuroimaging, most of which we still face today. Such challenges include (1) management of large volume and variety of forms in which the data are presented, (2) methods for the processing of brain images, (3) accessibility of data, and (4) the lack of access to neuroimaging results to investigators [2,3].

Among the most established neuroimaging databases is the Alzheimer's Disease Neuroimaging Initiative (ADNI) database [4], which currently contains data from over 1900 subjects, encompassing over 4000 MRI and PET scans, as well as clinical, cerebrospinal fluid, genetic, and biochemical biomarkers, which have been made available to researchers worldwide, who have made over 14 million downloads. Many other databases with more specialized audiences exist and have been cataloged in Neuroscience Information Framework [5]. These include the Minimal Interval Resonance Imaging in Alzheimer's Disease database [6], the OpenfMRI database [7], NeuroVault [8], The Virtual Brain [9], Neuroimaging Data Model [10], the Vanderbilt University Institute for Imaging Science Center for Computational Imaging XNAT (Extensible Neuroimaging Archive Toolkit-based repository) [11].

### Study Aims

NWSI serves as an automated, responsive, and scalable neuroimaging database solution. This new design serves also as a cyber physical system in that it offers users access to neuroimaging algorithms through the Internet and provides the needed computational resources with all the required processing, storage capabilities, security and operational maintenance. It comprises a Web interface and a set of replica Linux servers that perform specific tasks. Interacting with the system requires minimal computing knowledge, equivalent to what is expected from social media or similar type of Web interface. This interface would also serve the research community for applying their new data mining and deep learning concepts on a multimodal imaging platform [12].

NWSI is equipped with various useful tools such as Brain Extraction Tool (BET), brain image registration, image format conversion, data processing, and visualization mechanisms that help with rating and diagnosis. The current implementation includes (1) automatic quantification of volumes from anatomical MRIs, (2) 18F-Florbetapir and 18F-Florbetaben for Alzheimer disease (AD), (3) Fluorodeoxyglucose (FDG) PET analysis for epilepsy, and (4) DTI image processing for both AD and epilepsy. All the data results are collected in files and into a MySQL database and can be exported into both tabulated files and image files. The accumulated data can be used in future pipelines as input to multimodal and longitudinal studies.

NWSI utilizes an embedded, modified version of the Papaya viewer (a JavaScript medical research image viewer) developed by University of Texas Health Science Center. The viewer allows interactive display of cerebral regions, diffusion images, and PET data. All images are coregistered to the anatomical MRI as part of the pipeline; they can be displayed on the same viewer in stacked layers. Moreover, results have been validated by comparison with existing processed data, such as from the ADNI database, which provides an excellent source of raw and postprocessed data for validating the various functions of NWSI.

## Methods

### Data

Data included in NWSI involves both AD data and epilepsy data, which are recorded on a regular basis as more cases are routinely scheduled.

#### Alzheimer Disease Data

AD imaging data included in this Web interface were obtained from the 1Florida Alzheimer’s Disease Research Center (1Florida ADRC) and ADNI databases.

##### 1Florida Alzheimer’s Disease Research Center

NWSI was piloted using a database of MRI and amyloid PET images obtained through 1Florida ADRC. For this pilot project, currently 273 structural MRI, 43 18F-Florbetapir PET scans, and 89 18F-Florbetaben PET scans are available. The MRI images were obtained using a Siemens Medical System Skyra 3 Tesla Scanner. The DTI scans were used to measure radial, axial, and mean diffusivity, as well as fractional anisotropy (FA). PET images were obtained from a Siemens Biograph 16 Hi-Rez PET-CT machine.

##### Alzheimer's Disease Neuroimaging Initiative

Data used for validation purposes were obtained from the AD Neuroimaging Initiative (ADNI) database .The primary goal of ADNI has been to test whether serial MRI, 18F-Florbetapir PET, other biological markers, and clinical and neuropsychological assessment can be combined to measure the progression of mild cognitive impairment (MCI) and early AD. MRI scans were acquired from 1.5 T or 3 T scanners at multiple sites across United States and Canada.

#### Epilepsy Data

Epilepsy PET scans were obtained from Baptist Health South Florida, and Nicklaus Children's Hospital. There are currently 10 cases in the database, with more cases to be included in the near future as the schedule allows. The intent here is to include EEG data for 3D source localization in context with hypometabolism as observed through PET. Subjects from the Baptist Hospital of Miami were scanned with a Philips PET or computed tomography scanner with an FDG imaging agent. Subjects from Nicklaus Children's Hospital were scanned with a GE Discovery ST PET or CT system with an FDG imaging agent.

### Neuroimaging Web Services Interface and Hardware Architecture

#### Neuroimaging Web Services Interface

The Web interface driving NWSI is based on Drupal, a popular open source content management system, which is the platform for BBC, University of Oxford, the US Department of Energy, among other well-established organizations. Drupal provides a user-based platform, in which the core code for security and design tools are updated and patched frequently to address vulnerabilities, as well as to add new functionalities. New features can also be added to Drupal via modules that can be integrated with its core code, allowing new code to run on Drupal, while maintaining the core software secure and intact.

**Figure 1:**
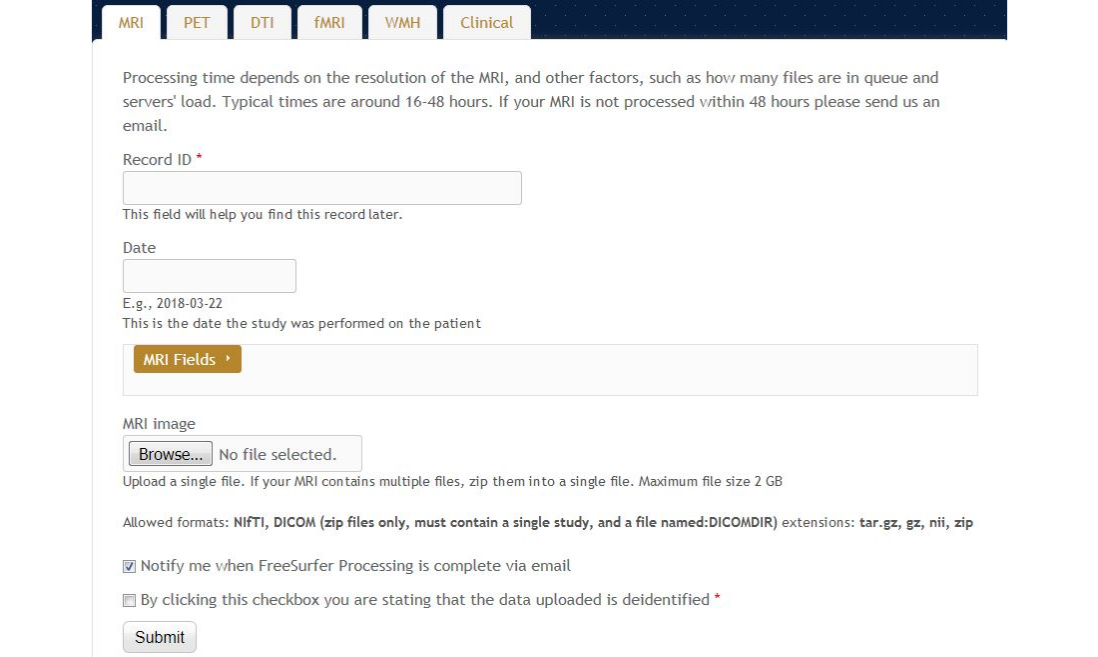
Data upload form, with magnetic resonance imaging (MRI) upload selected. Same function could be done with other modalities (positron emission tomography, diffusion tensor imaging, and functional magnetic resonance imaging).

The Web interface of NWSI has a simplified design, utilizing forms and uploaded files for most of the data input. Users of the interface, who are not familiar with Linux or its command line arguments, will be able to upload, view, and visualize existing data. Figure 1 shows the MRI upload form as an example. All data are deidentified before being uploaded to the server, and the user determines whether or not the data on NWSI will be shared with other users. Access to the site is provided through password-protected accounts.

Although the user interaction occurs through the Web interface, a set of replica servers (RSs), which run on Linux, perform a variety of asynchronous tasks, such as running FreeSurfer [13] on anatomical MRIs, or registering structural MRI to PET or DTI images. To keep the interface responsive, new tasks are sent to a work loader on the Web interface, which in turn sends tasks to one or more available RS(s) on the basis of their current status. Once the data is copied back to the Web server, the PostProcessing Core incorporates it into the system’s database and file system. The architecture is scalable, such that new RSs, which are easy to maintain clones with identical software, can be added on demand to the system.

The asynchronous communication between the Web interface and the RSs is achieved by securely copying files. Some of these files are data to be processed, whereas others are status reports and workload balance data. MRI, PET, DTI, and fMRI images are processed on the RSs, but smaller tasks, such as brain extraction or registration, are done synchronously on the Web server by the Short Task Module. Tasks selected to run on the Small Task Modules must be brief, no longer than few minutes in duration to keep the Web server responsive.

The interface is illustrated in a PowerPoint presentation and video included in Multimedia Appendix 1 and Multimedia Appendix 2, respectively.

#### Neuroimaging Web Services Interface Hardware Architecture

**Figure 2:**
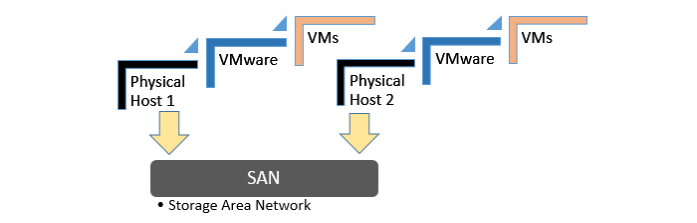
Virtual architecture.

Virtual technology and Modular Smart Array Systems are used to host the NWSI Web interfaces and its RSs. The cluster-aware infrastructure has two ProLiant DL 380 G7 servers, forming a centralized pool of resources that is used to create virtual machines (VMs) which run their own operating system (OS). Figure 2 illustrates the virtual architecture of NWSI. The Web interface runs on a VM running an Ubuntu open source software OS, Apache software, and a MySQL database management system. Since the processing time for a single MRI ranges from 8 to 12 hours, as many as 16 MRIs can be processed simultaneously, using RSs with 8 cores each. PETs and DTIs are processed in 15 and 10 min, respectively. The use of a virtual-server environment adds availability, security, and scalability to the Neuroimaging Web-interface application.

**Figure 3:**
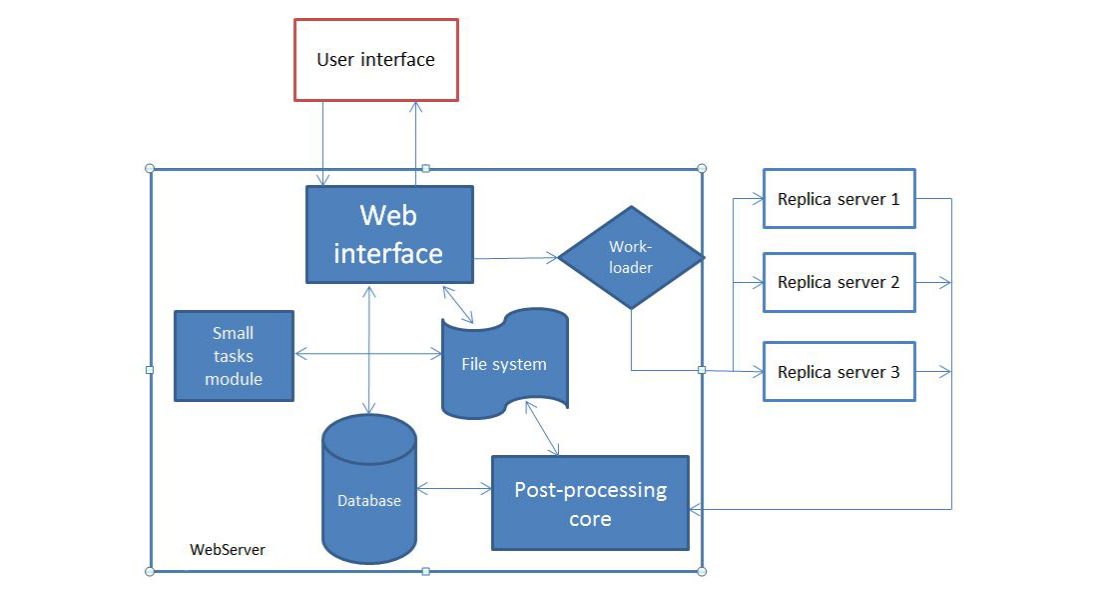
Architecture of the Neuroimaging Web Interface System.

Furthermore, new VMs can be added to the current design to increase the capability and performance of the system. RSs can reside in a private cloud or the Internet, as long as files can be securely copied between them and the Web interface. The Web interface can be accessed anywhere in the world with a fast Internet connection and a browser. It is both device and OS independent. The Drupal theme is responsive and tablet or phone friendly. Figure 3 shows the architecture of the neuroimaging Web-interface system, wherein the user interacts with the Web server through the Web browser.

Results from completed tasks are readily viewable. New tasks are sent by the Work loader, to an available RS, which sends the completed task to the PostProcessing Core, from which new values are entered into the database and raw and new images are stored on the file system. Registration, Brain Extraction, and other smaller tasks are processed on the Web server by the Small Tasks Module.

### Image Viewer

Papaya, developed by the University of Texas Health Science Center at San Antonio, is a powerful open source, interactive, JavaScript-based image viewer that is incorporated within NWSI. The Web interface accepts the 3 most common formats as input—DICOM, ANALYZE, and NIfTI [14], but converts all files to NIfTI, which is versatile, more compact, and widely used. It is noted that supporting software, such as FSL [15], only accepts NIfTI as input.

**Figure 4:**
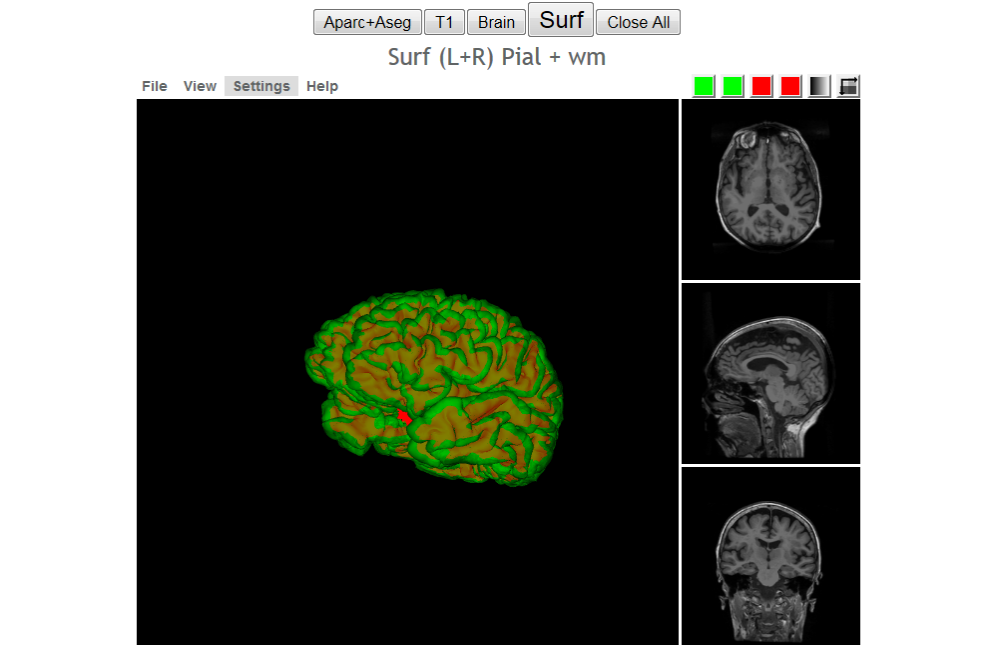
Interactive viewer, showing the surface reconstruction of and anatomical magnetic resonance imaging (MRI), Green represents the gray matter, Red represents the white matter.

**Figure 5:**
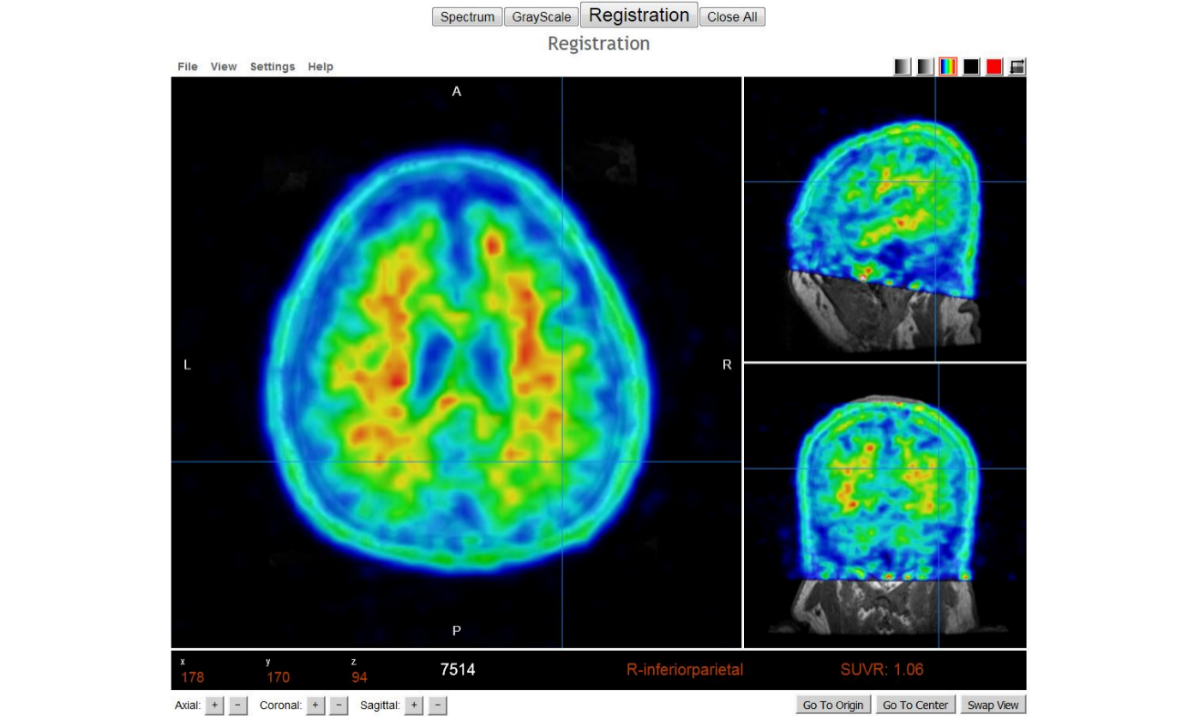
Image shows positron emission tomography (PET) viewer’s full interface.

**Figure 6:**
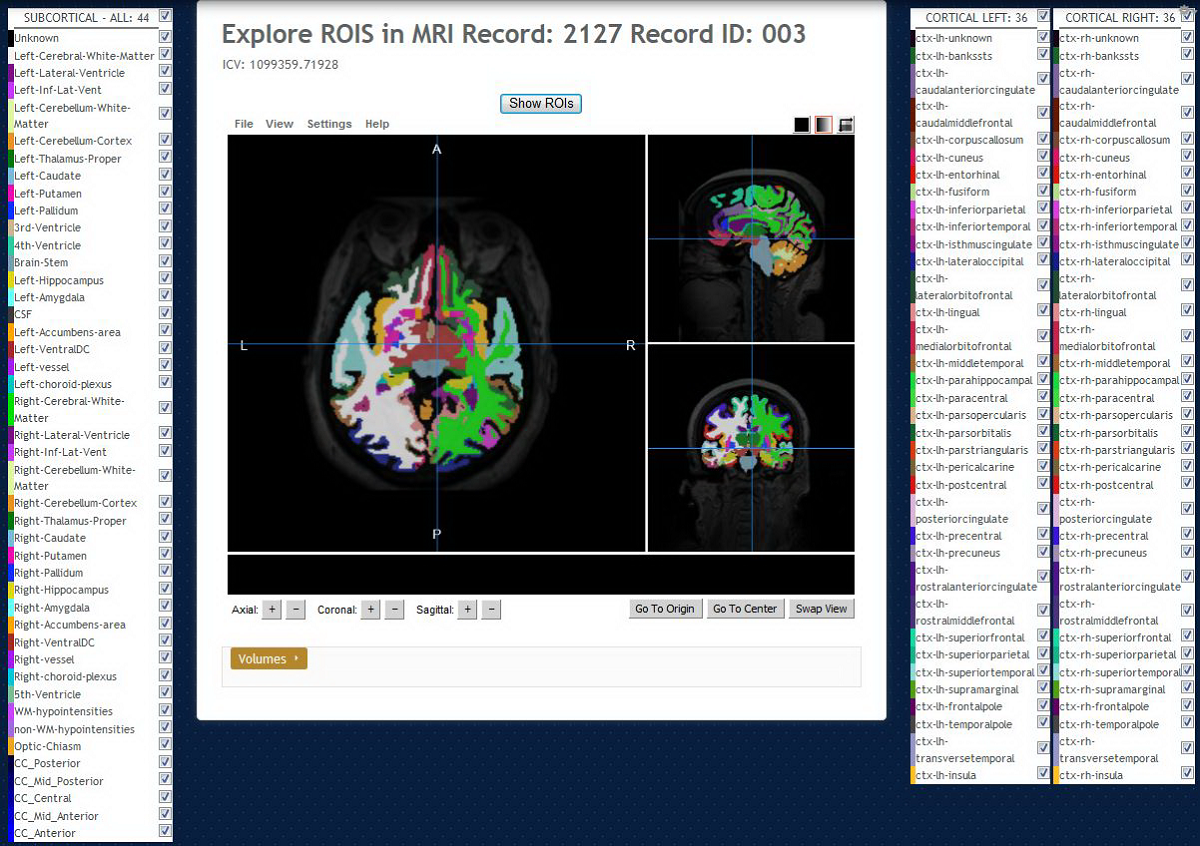
Regions of interest (ROI) Explorer: visualizing FreeSurfer segmentation. By default, all regions, cortical and subcortical, are shown.

**Figure 7:**
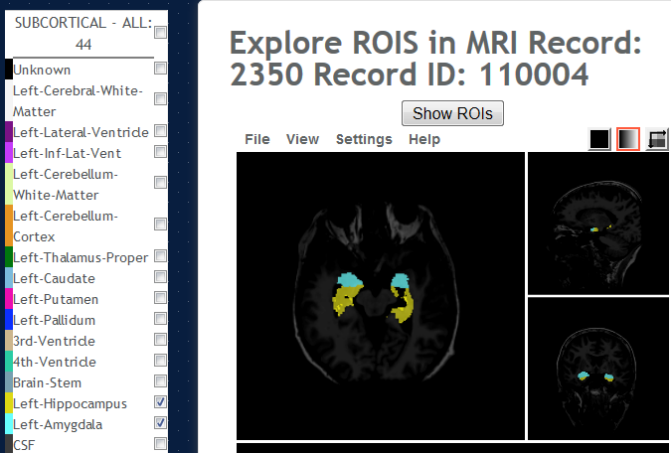
Regions of interest (ROI) Explorer: showing the selected subcortical left and right-hippocampus and amygdala regions.

The version of Papaya in NWSI has been modified to display FreeSurfer labels and custom color tables. The Web interface is also capable of displaying specific color-coded FreeSurfer regions; whole brain segmentation; interactive surfaces; and PET, fMRI, and DTI images. As part of our processing pipeline, all image files are registered to the structural MRI scan, making it possible to display several modalities as layers in the same viewer. Among the tools embedded on the Web interface and available to the user by Papaya are color selection, a measuring tool, an axis viewer, and an image transparency modification tool, all of which are standard in many other viewers. The user can display these images online without having to save any files to the local hard disk (see Figures 4 and 5 below for illustrative examples). Furthermore, the ROI explorer page displays a color-coded segmentation of FreeSurfer regions. This is especially useful for researchers who are not familiar with FreeSurfer labels, but are familiar with the human brain anatomy. Pertinent information can be visualized by clicking on specific regions to scrutinize what the different regions reveal (see Figures 6 and 7).

## Results

### Volumetric and Cortical Thickness Calculations From Anatomical Magnetic Resonance Imaging

The basic functionality of NWSI depends on using FreeSurfer to reconstruct cortical surface models (gray-white boundary surface and pial surface) from structural MRIs and to output regional cortical and subcortical volumes, cortical thickness, and other values derived from input image segmentation (see FreeSurferWiki or a complete list of such measures). FreeSurfer also outputs image files that define the segmentation and replaces intensity on these files by numeric values representing the segmented regions. These files are used in PET standardized uptake values (SUV) calculations.

NWSI processes all structural MRIs on a local server, ensuring that the FreeSurfer results are validated and not affected by the OS version adopted [16]. FreeSurfer specifies in *fswiki* that certain OS-level libraries might affect the results. Thus, as new RSs are added to the system, it is imperative to test them before deployment to make sure the results are validated against established ones.

**Figure 8:**
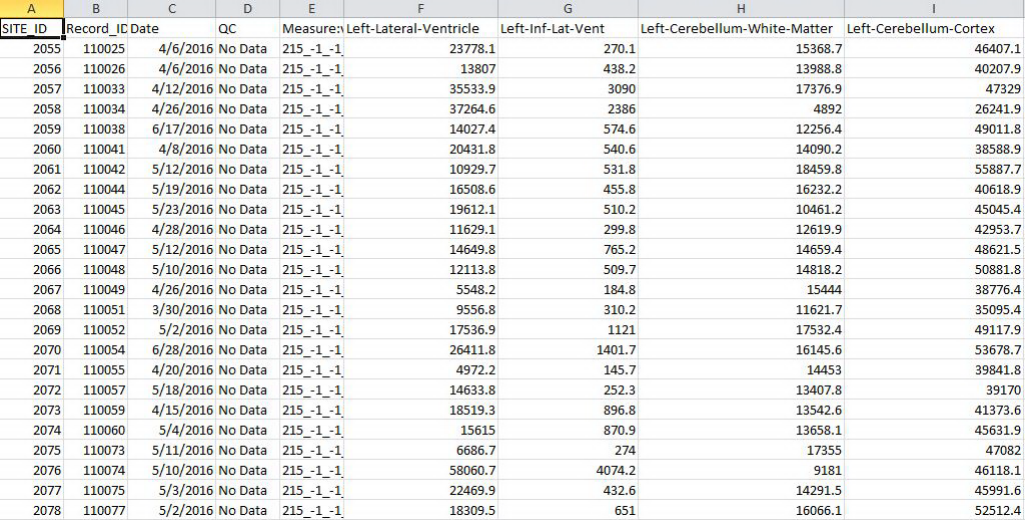
Sample tabulated output for subcortical regions.

An important issue, which is resolved by NWSI, is in the ability of merging of data from different sources, which is a nontrivial task due to factors such as scanner bias, scanner field strength, etc [17]. This problem is best addressed by processing all values with the same hardware and software. Consequently, subjects from one institution can be merged with subjects from another institution, and the results can be downloaded and tabulated in a format as shown in Figure 8. In fact, NWSI has managed to achieve that by combining subjects from our own 1Florida ADRC to subjects from the ADNI database.

#### Quality Control

Quality control within NWSI is performed by visual inspection of the output image files, similar to the process used by ADNI. The image viewer can be used to inspect the image to determine whether segmentation of the structural image by FreeSurfer was subject to errors, such as truncated sections of the structural image, poor resolution, or contamination by noise in the system. In cases of such errors, it is still possible to isolate and study only those regions that segmented correctly. Depending on the outcome, each MRI is catalogued by Quality Control as Fail, Pass, Hippocampus-only, or Partial. Many studies implement normalization using global values, such as the intracranial volume, derived from FreeSurfer. If an image receives a Fail grade, it is not possible to include it in studies that depend on global values.

#### 18F-Florbetapir Positron Emission Tomography or 18F-Florbetaben Positron Emission Tomography Analysis

**Figure 9:**
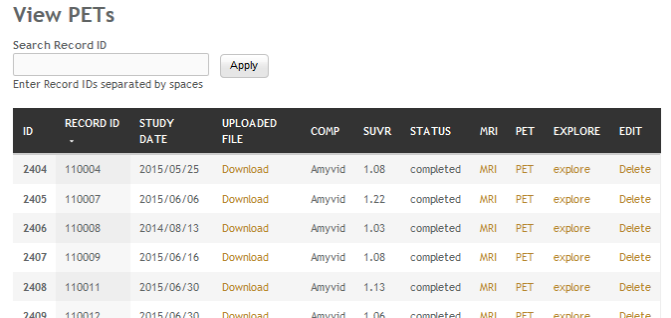
Page listing all positron emission tomographies (PETs) in the account, with links to the magnetic resonance imaging (MRI) used as reference.

**Figure 10:**
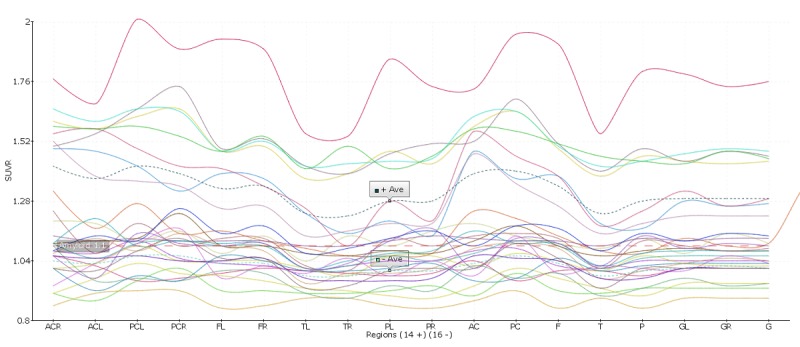
Graph showing the Standardized uptake value ratio (SUVR) distribution of all 18F-Florbetapir positron emission tomographies (PETs) uploaded by a user.

**Figure 11:**
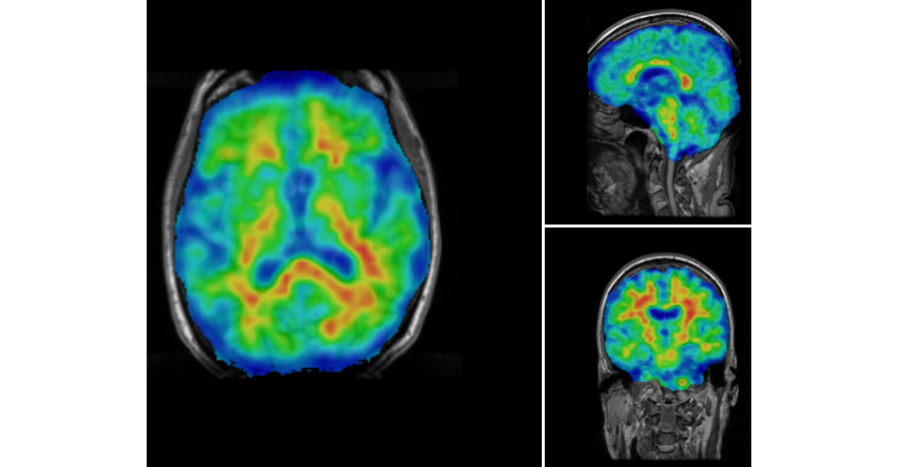
Higher concentration of 18F-Florbetapir shown in warmer colors of the spectrum look up Table (LUT).

**Figure 12:**
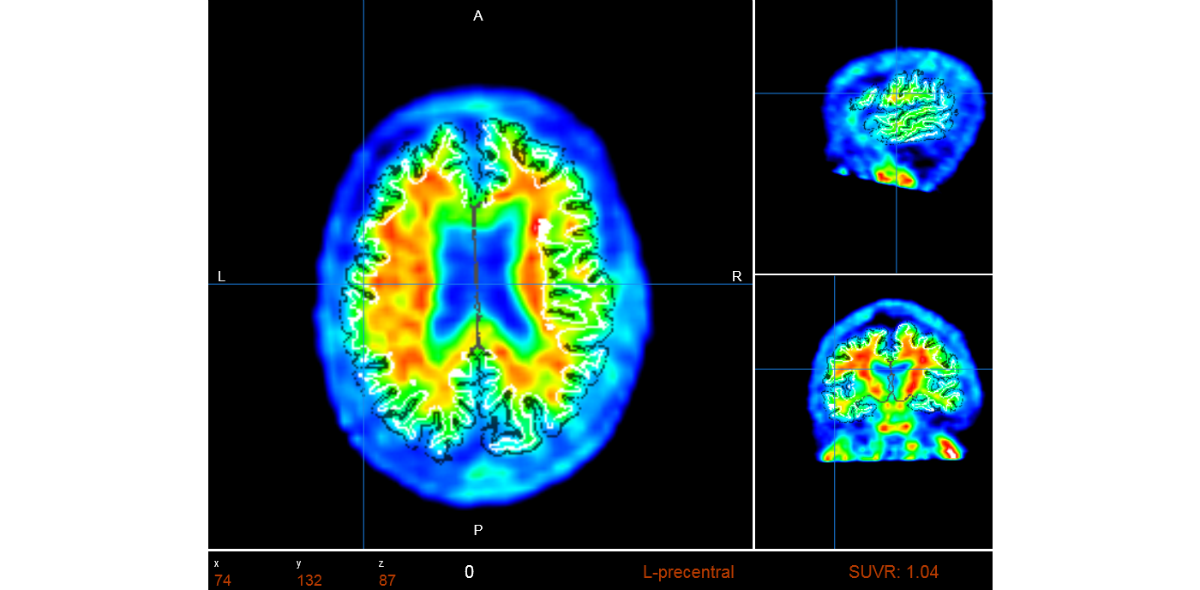
Positron emission tomography (PET) image is overlaid with the white matter surface (shown in White) and gray matter surface (shown in Black). Selected region’s standardized uptake value ratio (SUVR) and name of region are displayed in Red at the bottom of the screen.

**Figure 13:**
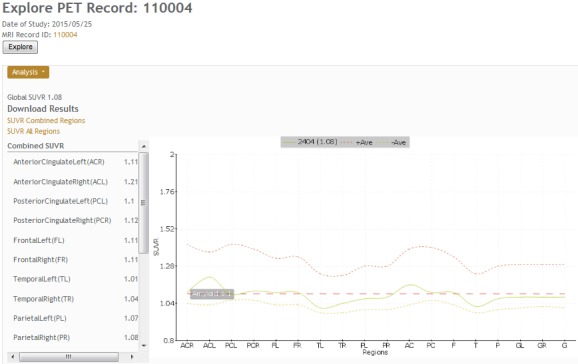
Positron emission tomography (PET) results page, which shows all standardized uptake value ratio (SUVR) values, links to download tabulated results, and a graph comparing the result to the averages determined in the system.

Quantification of regions of interest (ROI) is still defined manually, but automatic standardized uptake value ratio (SUVR) calculations and segmentation of PET images have become the gold standard [18]. NWSI implements several PET analysis pipelines for FDG and 18F-Florbetapir images. Before uploading a PET scan, a related structural MRI must already exist in the system; the user is presented with a form in which an existing MRI must be selected. After the PET scan is uploaded, it is copied to one of the RSs for processing and can be accessed through a form that lists all uploaded records, as shown in Figure 9. This form also contains graphs showing the distribution of all PET scans uploaded by the user as shown in Figure 10. Once a PET scan is processed, it can be displayed on the interactive image viewer, as shown in Figure 11 and with white and gray matter surfaces segmented as shown in Figure 12, and then quantitative data can be downloaded from the PET scan page, as shown in Figure 13.

#### Magnetic Resonance Imaging-Positron Emission Tomography Image Registration

**Figure 14:**
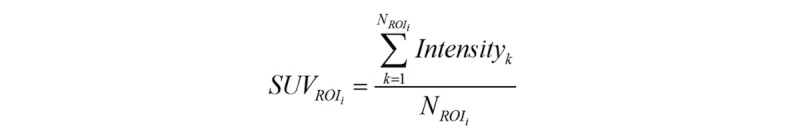
Formula to calculate the SUV value. N(ROI) is the total number of voxels in ROI(i) , and Intensity K is the value of voxel k in ROI (i) in PET. SUV: standardized uptake value; ROI: region of interest; PET: positron emission tomography.

**Figure 15:**
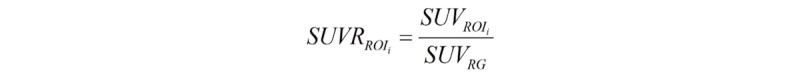
Formula to calculate the regional SUVR values, by normalizing with SUV of reference region. SUVR: standardized uptake value ratio.

**Figure 16:**
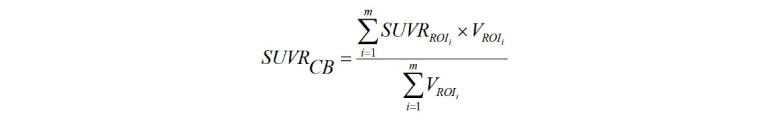
Formula to calculate the SUVR value of combined regions. CB represents combined regions, and V (ROI(i)) is the volume of the region. SUVR: standardized uptake value ratio; ROI: region of interest.

It is essential that the PET image is coregistered to the anatomical MRI since all calculations depend on how closely the anatomical regions of the 2 images overlap. A particular challenge for aging and AD and other neurodegenerative diseases is the issue of atrophy correction. It should be indicated that coregistration to MRI images largely reduces artifacts related to atrophy. The current implementation aligns the images using FSL. The alignment uses 12 degrees of freedom (3 translations, 3 rotations, 3 scalings, and 3 Skews or Shears). Then, a custom R script (R is an open source statistics software developed by Bell Laboratories) opens the coregistered PET and the FreeSurfer segmentation volume, performing a voxel by voxel analysis of the intensities of the PET file and accumulating the values per anatomical ROI. In relation to these SUVR graphs, it is noted that the average over a region provides the *SUV* value over that region of interest (*ROI*) as given in Figure 14. The SUVR values are then determined by dividing the *SUV*_ROI_ by the SUV of a region of reference (*SUV*_RG_) as expressed in Figure 15. Larger regions, aggregated from FreeSurfer subregions can also be calculated. SUVR, for combined FreeSurfer regions (CB), are calculated by a volume-weighted average of previously calculated SUVR as described in Figure 16.

Current literature reveals the merits of using an assortment of reference regions, such as the total or eroded subcortical white matter, the brain stem, the whole cerebellum, or the cerebellar white matter. SUVR results, normalized by the whole cerebellum, have been validated using the values reported by ADNI. NWSI also calculates SUVR using unilateral or bilateral cerebellar white matter. These values can be exported on tabulated files. PET imaging with 18F-Florbetaben also measures global cortical amyloid load and uses a similar processing pipeline to 18F-Florbetapir images. However, statistics and results on NWSI are reported separately to avoid bias. Studies have shown that there are no marked differences in the diagnostic accuracy of the amyloid binding ligand.

#### Fluorodeoxyglucose-Positron Emission Tomography in Epilepsy

**Figure 17:**
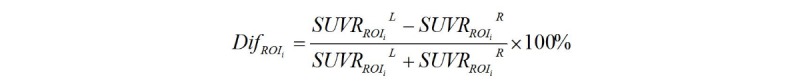
Formula to calculate the difference between the standardized uptake value ratio (SUVR) of bilateral regions. L and R represent Right and Left hemisphere, respectively.

PET imaging using FDG, labeled with a positron emitting tracer (Fluorine-18), or FDG-PET, is used to measure regional glucose metabolism, which is strongly correlated to the regional neuronal activity in the brain [19]. To study epileptic conditions using FDG-PET, in combination with structural MRI, regional SUVR is calculated using the cerebellar white matter, or the whole cerebellum, as a reference region [20]. In studying epilepsy, special consideration needs to be given to identifying focal conditions in one hemisphere and to account for surgical resection of regions in the brain. The FDG-PET pipeline in NWSI allows a choice of several reference regions, including the whole cerebellum, the cerebellar white matter, the average of all bilateral cortical regions (global cortical SUV), or all regions for a single intact hemisphere, especially in subjects who have had a prior resection in one hemisphere. The PET images are superimposed on MRI brain scans for defining the underlying structure and brain regions which have been resected. Regional SUVR is derived similar to the procedure for amyloid PET scans, and asymmetry in corresponding bilateral regions is calculated by dividing the difference in SUVR among corresponding bilateral regions by their sum and multiplying by 100%, as shown in Figure 17. Reference regions are not required in this calculation of asymmetry; a difference of 10% or greater between bilateral regions is typically considered to be consequential*.*

#### Diffusion Tensor Imaging

**Figure 18:**
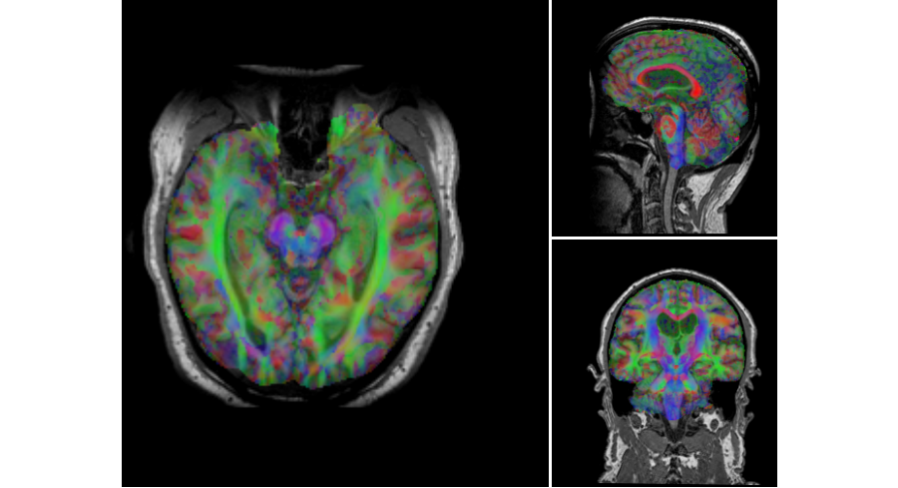
Sample processed Alzheimer’s disease (AD) diffusion tensor imaging (DTI). V1 modulated by fractional anisotropy (FA). Colors represent direction of water movement: Green is front to back. Blue is head to foot. Red is left to right.

**Figure 19:**
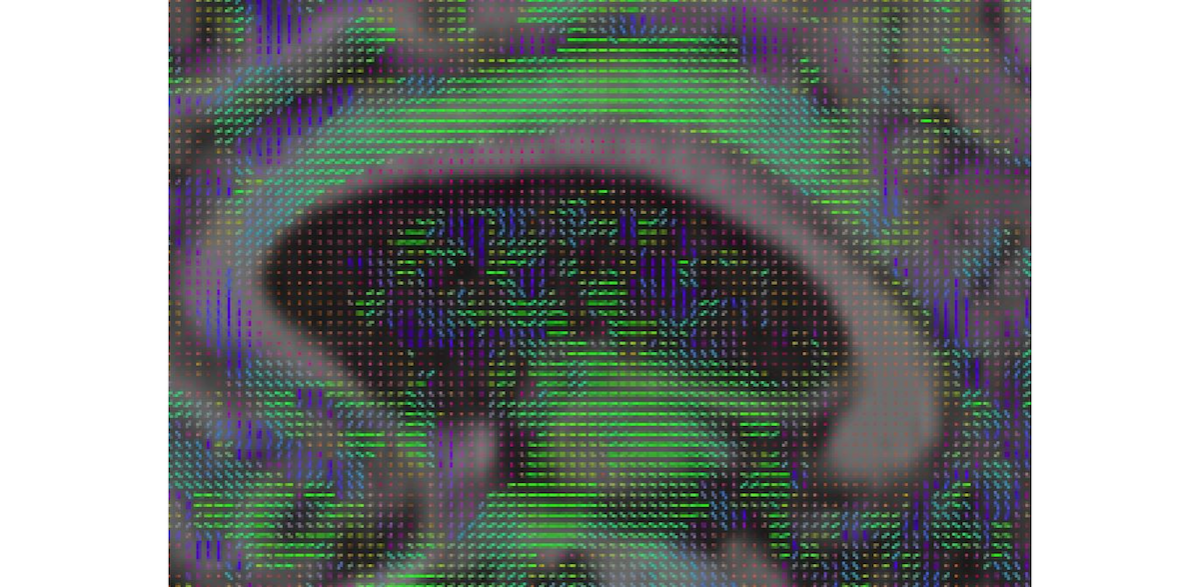
Close up of Diffusion Tensor Imaging (DTI) image showing the V1 eigenvectors pointing at the direction of water diffusion around the ventricle.

DTI analysis on NWSI is still under development. Currently, DTI is obtained by executing the DTIFit FSL tool on an anatomically coregistered diffusion-weighted image (DWI) that has been corrected for Eddy currents. The DTIFit program fits a diffusion tensor model at each voxel. The resulting DTI eigenvalues and related eigenvectors, which reflect the direction of water movement and diffusion properties of a tissue, can be shown on the Web interface viewer modulated by the FA image. DTI is obtained from DWIs. A diffusion tensor is calculated for each voxel (3×3 matrix). The direction of the fibers is indicated by the tensor’s eigenvector. The images are color coded, with Red, Blue, and Green; indicating right to left, foot to head, and anterior to posterior directions, respectively. As these images are coregistered to the anatomical MRI, further analysis can be done to obtain numeric values for anatomical ROIs (see Figures 18 and 19).

#### Data Convert, Registration, and Brain Extraction Tools

**Figure 20:**
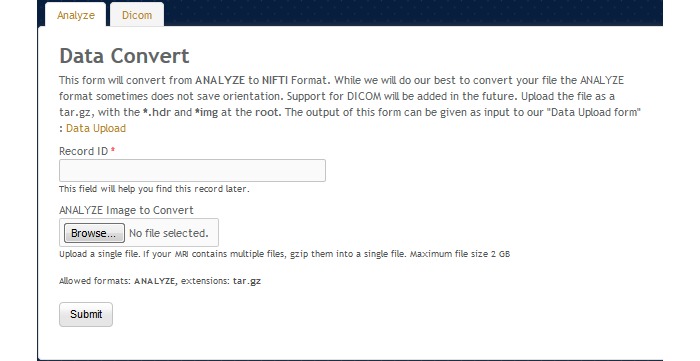
The data convert tool.

NWSI offers an assortment of other tools. The Data Convert (DC) tool, as shown in Figure 20, provides an interface to convert from DICOM and ANALYZE to NIfTI format. The DC tool also handles image compression, image orientation, and other issues which arise from transfer syntax in DICOM images. The interface is simple to use; it asks the user to upload a file and provides an identification field (record ID) and then converts the file to NIfTI. The output result can be downloaded and used as input to other forms within the site.

#### The CoRegistration Tool

This tool coregisters one brain image to another, from similar or different modalities. This form uses FSL tools to align the images and exports many of the options to the user, such as (1) extract the brain from source images before registration and (2) define the degrees of freedom, cost function, or angle to rotate the images. For most of the AD data processed in NWSI, default FSL registration parameters work well. However, some images have noise or artifacts and cannot be used for registration. CRT allows the user to find the registration parameters for individual images before they are uploaded to other processing pipelines. Current and previous results can be inspected in an embedded viewer. Coregistration is the main step to many processing pipelines, especially for multimodal imaging.

#### The Brain Extraction Tool

This tool interfaces with FSL and is extremely useful for extracting the brain structure from an image in any modality.

### Evaluation and Validation

#### Magnetic Resonance Imaging Values

As cautioned earlier, FreeSurfer values obtained from the same FreeSurfer version can vary among different hardware and software platforms. Hence, for this proposed Web interface, the RSs are calibrated, making sure they always provide the same results before their deployment. ADNI renders FreeSurfer results calculated on FreeSurfer 5.1. In order to validate NWSI, a paired *t* test was performed to compare the values reported in the ADNIMerge file and the NWSI results. A total of 20 ADNI cases were selected at random from the 4 main diagnoses (AD; early mild cognitive impairment, EMCI; late MCI; and cognitively normal). One of the selected subjects failed ADNI’s quality control for the Mid Temporal region, but it was successfully processed by NWSI. Table 1 shows the validation of the NWSI results in comparison with ADNI data, with ADNI being the gold standard for Alzheimer’s MRI analysis.

**Table 1:** Magnetic Resonance Imaging (MRI) processing comparison by Neuroimaging Web services interface (NWSI) quality control.

Region	NWSI^a^ FreeSurfer 5.3	ADNI^b^ Merge FreeSurfer 5.1	Difference (%)	Pearson correlation coefficient	Paired *t* test^c^
Hippocampus	7041 (SD 529)	7043 (SD 1404)	−3.00	.99 *(P*<*.* 001)	−0.03 (.9788)
Entorhinal cortex	3252 (SD 717)	3504 (SD 827)	−6.50	.88 (*P*<.001)	−2.8 (.01)
Middle temporal	19040	20020	−3.40	.97 (*P*<.001)	−5.3 (.0001)
Intracranial volume	1486538 (SD 154661)	1482872 (SD 152534)	2.40	.99 (*P*<.001)	−1.0 (0.33)

^a^NWFI: Neuroimaging Web Services Interface.

^b^ADNI: Alzheimer's Disease Neuroimaging Initiative.

^c^H0: Difference=0.

**Table 2:** Paired *t* test comparing Neuroimaging Web Services Interface (NWSI) and Alzheimer's Disease Neuroimaging Initiative (ADNI) Merge 18F-Florbetapir Positron Emission Tomography (PET) Global standardized uptake value ratio (SUVR) values.

Results	NWSI^a^	ADNIMERGE^b^
Mean	1.22	1.217
Variance (SUVR^c^)	.047	.044
Observations	18	18
*t* Stat(degrees of freedom 17)	.26	—
*P* (T≤*t*) two-tail	.79	—
*t* critical two-tail (SUVR)	2.11	—

^a^NWSI: Neuroimaging Web Services Interface.

^b^ADNI: Alzheimer's Disease Neuroimaging Initiative.

^c^SUVR: standardized uptake value ratio.

ADNIMerge and NWSI FreeSurfer results are highly correlated. There is a small statistical difference for Mid Temporal and Entorhinal cortex. ADNIMerge was processed with FS5.1, and NWSI uses FS5.3. FreeSurfer 5.3 was a major upgrade to 5.1. Different FreeSurfer versions produce different results, but it does not imply lack of validity. Classification results can still be reliably used [21].

#### 18F-Florbetapir Positron Emission Tomography

NWSI PET SUVR values have been validated with the values on ADNI data as reported by Jagust [22]. All ADNI PET results use 18F-Florbetapir. A random selection of 20 subjects from ADNI was used. Native PETs and MRIs were processed in NWSI, and the calculated global SUVR values were compared with the values reported by ADNI. Table 2 shows the paired *t* test results for comparing FLORBETAPIR (a PET radiopharmaceutical compound used for AD diagnostic) PET data obtained in NWSI, generated from ADNI cases, to the results provided by ADNI. This 2-tailed *t* test shows no statistical significance (*t* Stat<*t* Critical 2 tails) as can be seen from Table 2.

### Cost

NWSI scalability allows a large number of additional RSs. The basic requirement is 2 servers—one hosting the Web interface and the other for processing images. This setup was the initial prototype which worked effectively for small batches of less than 20 or 30 sets uploaded simultaneously. The cost of maintaining 2 dedicated servers is low; services such as GoDaddy Operating Company provide each server from US $69.99 per month for a dedicated Linux server. The current prototype is installed on a distributed system located at Florida International University. This type of setup is more secure and easier to manage, and has a larger price tag. FIU (Florida International University) paid US $62,000, including hardware and software licenses. If all the resources are utilized, the FIU setup can service many requests, with the capability to process hundreds of MRIs and PETs per day. The prototype only uses 3 servers from up to 15 servers which can be created on this distributed system with 8 cores and 32 GB each.

## Discussion

### Principal Findings

The feedback provided by medical doctors (MDs) and other researchers has been invaluable. The system, as currently developed, is the result of many hours dedicated to understanding the domain of neuroimaging and the needs and requirements of MDs. The medical images displayed in the Papaya viewer contain layers defining the edge of the gray and white matter, custom LUTs created based on how MDs visualize the specific results, and many other enhancements that emerged from interacting with the different medical teams who have used NWSI.

Data entry into NWSI has been evaluated based on user feedback. The forms, as explained before, are intuitive and similar to many other forms on the Internet. Uploading an MRI into NWSI is as simple as updating a Facebook or Instagram status.

New studies are added every month to NWSI. As more data is uploaded, it is possible to create methods for merging similar data from different sources. This allows, for example, using the control scans from one account to enhance another study lacking controls. Multimodal pipelines can also be created based on merging PET, structural and functional MRI, and DTI. The current implementation did not include fMRI, but our colleagues at the University of Florida will be processing all resting state fMRIs to be included in this Web interface, and the DTI processing is currently limited to few cases, but the intent is to enhance this work with more cases to be processed over the next year. Expanding those pipelines will allow multimodal pipelines to be created for enhanced multimodal studies. New processing pipelines can be exposed to the user, allowing inclusion of previously processed cases and broadening the scope of new studies.

FreeSurfer and FSL were the natural choices for segmentation and registration, especially since ADNI data were readily available and already processed using the well-established FreeSurfer and FSL software. There are other software packages that also provide excellent results, such as 3D Slicer (an open source platform for medical image informatics developed by an international community), AFNI (Analysis of Functional NeuroImages: a set of programs for processing, analyzing, and displaying functional MRI data, developed by the National Institute of Health), and SPM (Statistical Parametric Mapping is a software designed for the analysis of brain imaging data sequences, developed by members and collaborators of the Wellcome Trust Centre for Neuroimaging). For future development, it will be possible to add pipelines employing these software packages and for the user to select which one to use at the upload-form phase.

### Conclusions

NWSI provides a platform for storing and processing neuroimaging data. All data are deidentified before being uploaded to the server. NWSI is accessible worldwide. The user interacts with the processing pipelines through a simple interactive Web interface, which allows the users to upload and process images of the brain and view the results directly on the browser. The multiuser interface allows privacy among researchers, as well as data sharing. Data are protected on the secured server, whereas communication with the user is encrypted. Pipelines that process structural MRI and amyloid PET scans have been validated with existing and well-established databases such as ADNI. NWSI stores all results in SQL tables and files, facilitating the selection and processing of existing data into new pipelines. As such, NWSI offers a complete solution for neuroimaging studies with multiuser tools for data processing and visualization, as well as for downloading to other platforms for further processing.

## Appendix

Multimedia Appendix 1 Slideshow presentation describing the system.

Multimedia Appendix 2 Video of system description.

## Abbreviations

AD: Alzheimer disease
ADNI: Alzheimer’s Disease Neuroimaging Initiative
BET: Brain Extraction Tool
CB: Combined FreeSurfer Regions
CT: computed tomography
DC: Data Convert
DTI: Diffusion Tensor Imaging
DWI: Diffusion-Weighted Image
EMCI: early mild cognitive impairment
FA: fractional anisotropy
FDG: fluorodeoxyglucose
fMRI: functional magnetic resonance imaging
fswiki: FreeSurferWiki
MCI: mild cognitive impairment
MD: medical doctor
MRI: magnetic resonance imaging
NWSI: Neuroimaging Web Services Interface
OS: operating system
PET: positron emission tomography
ROI: regions of interest
RS: replica server
SUV: standardized uptake values
SUVR: standardized uptake value ratio
VM: virtual machine
1Florida ADRC: 1Florida Alzheimer’s Disease Research Center
