# Supplementary material for: A Neuroimaging Web Services Interface as a Cyber Physical System for Medical Imaging and Data Management in Brain Research: Design Study
Source: JMIR Med Inform. 2018 Apr 26;6(2):e26. doi: 10.2196/medinform.9063 (PMC5945984; doi:10.2196/medinform.9063)
Supplement: Multimedia Appendix 1 [file medinform_v6i2e26_app1.pptx]

## Slide 1
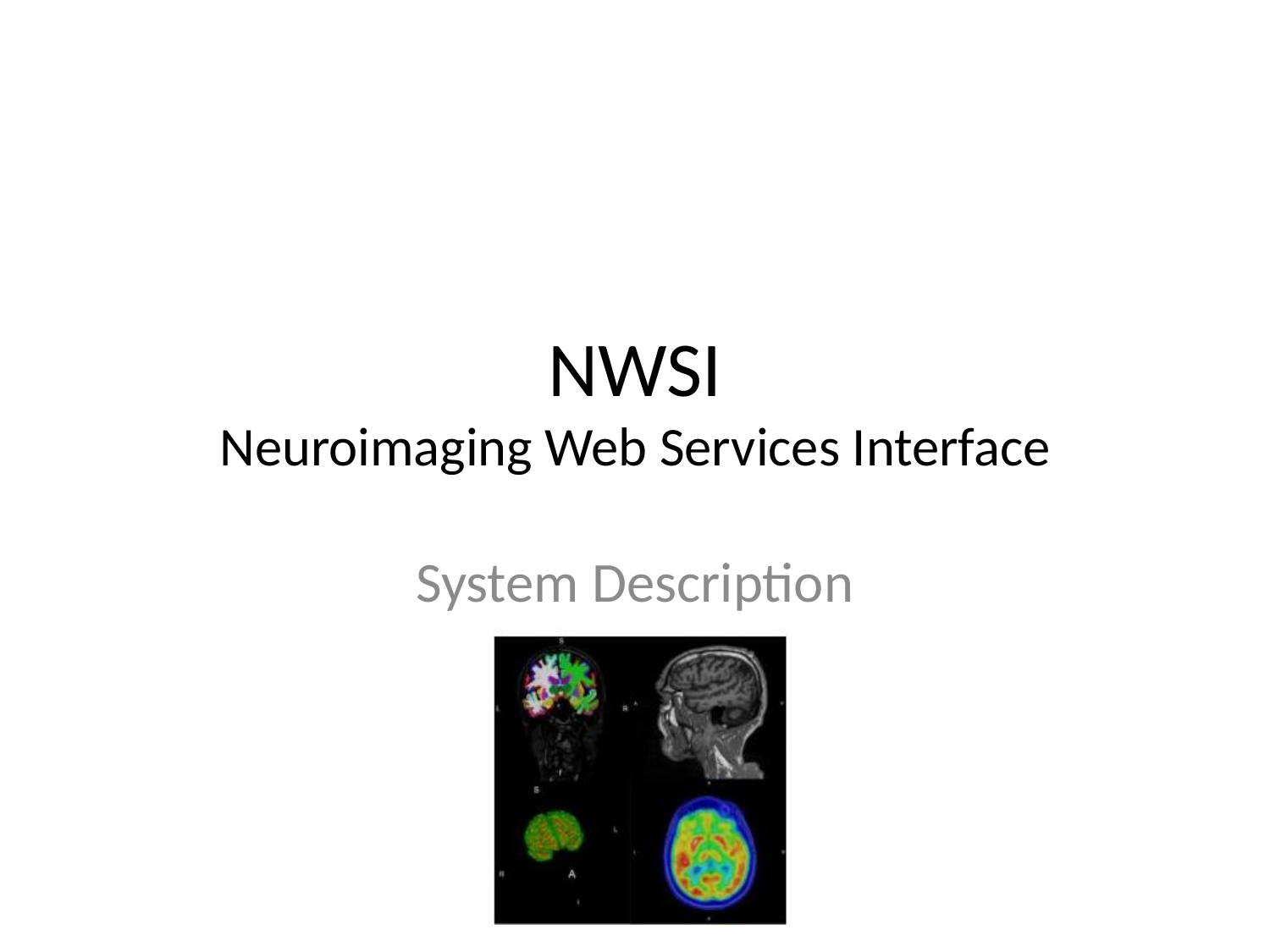

# NWSI Neuroimaging Web Services Interface
System Description

## Slide 2
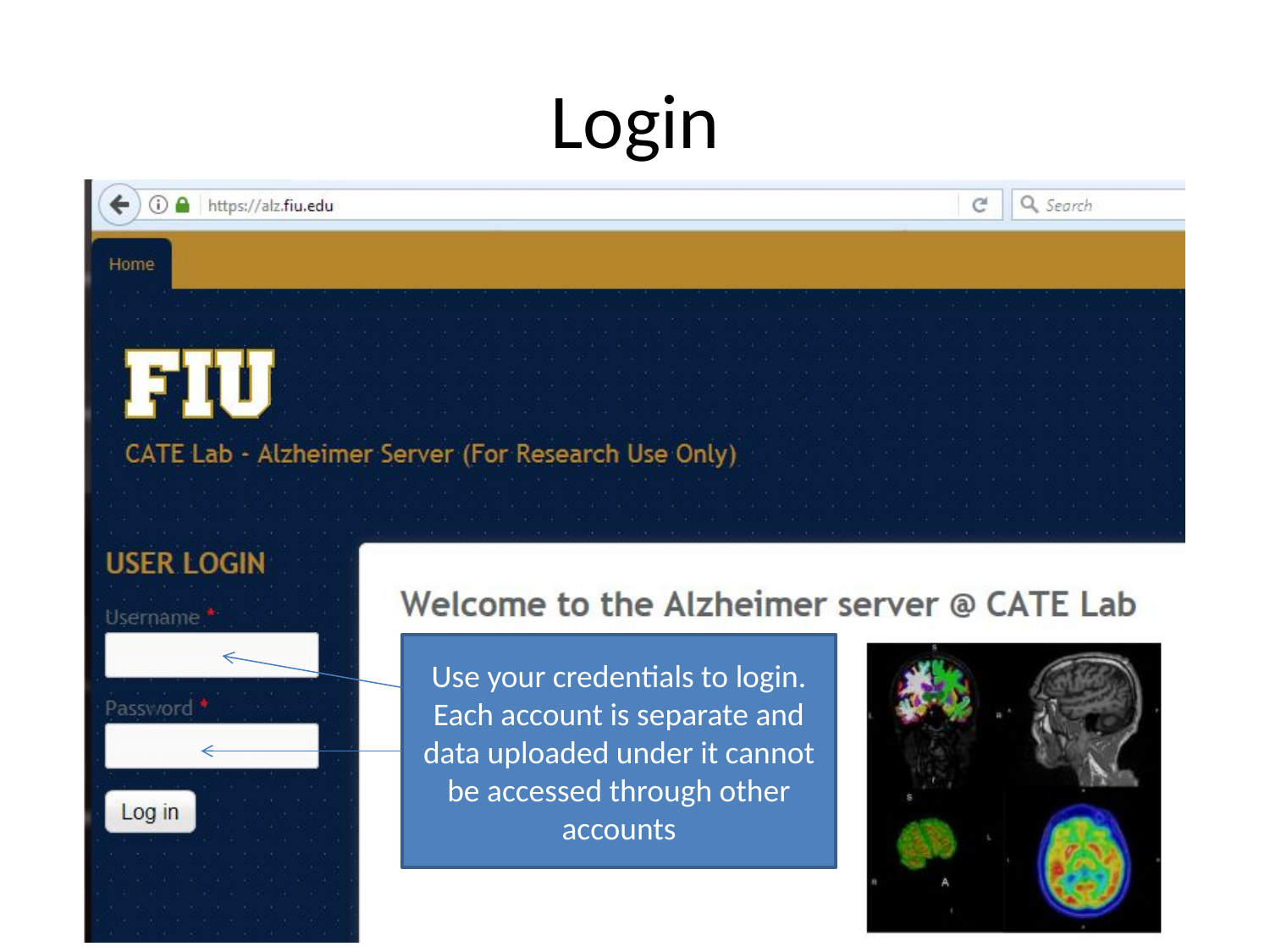

# Login
Use your credentials to login. Each account is separate and data uploaded under it cannot be accessed through other accounts

## Slide 3
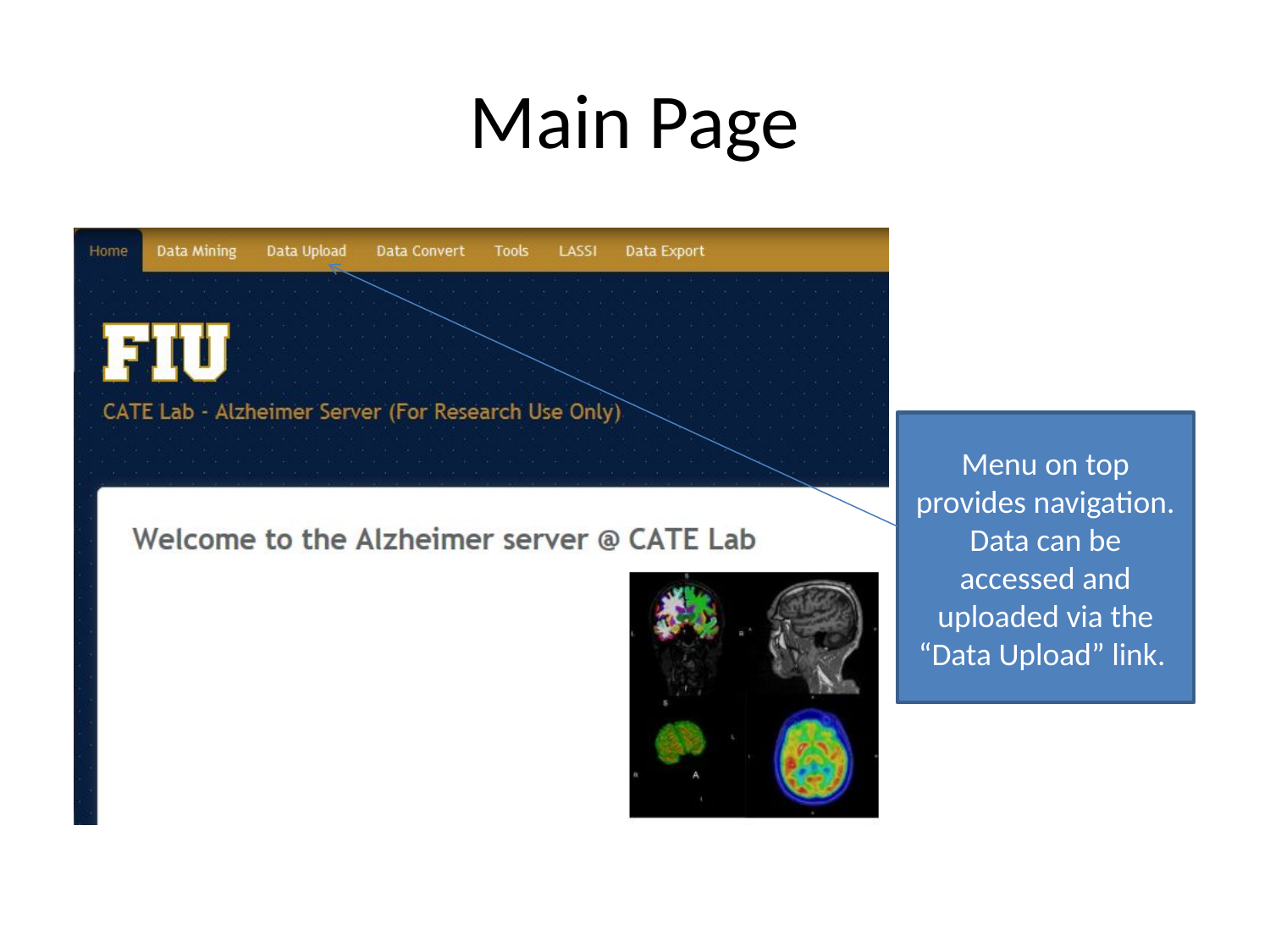

# Main Page
Menu on top provides navigation. Data can be accessed and uploaded via the “Data Upload” link.

## Slide 4
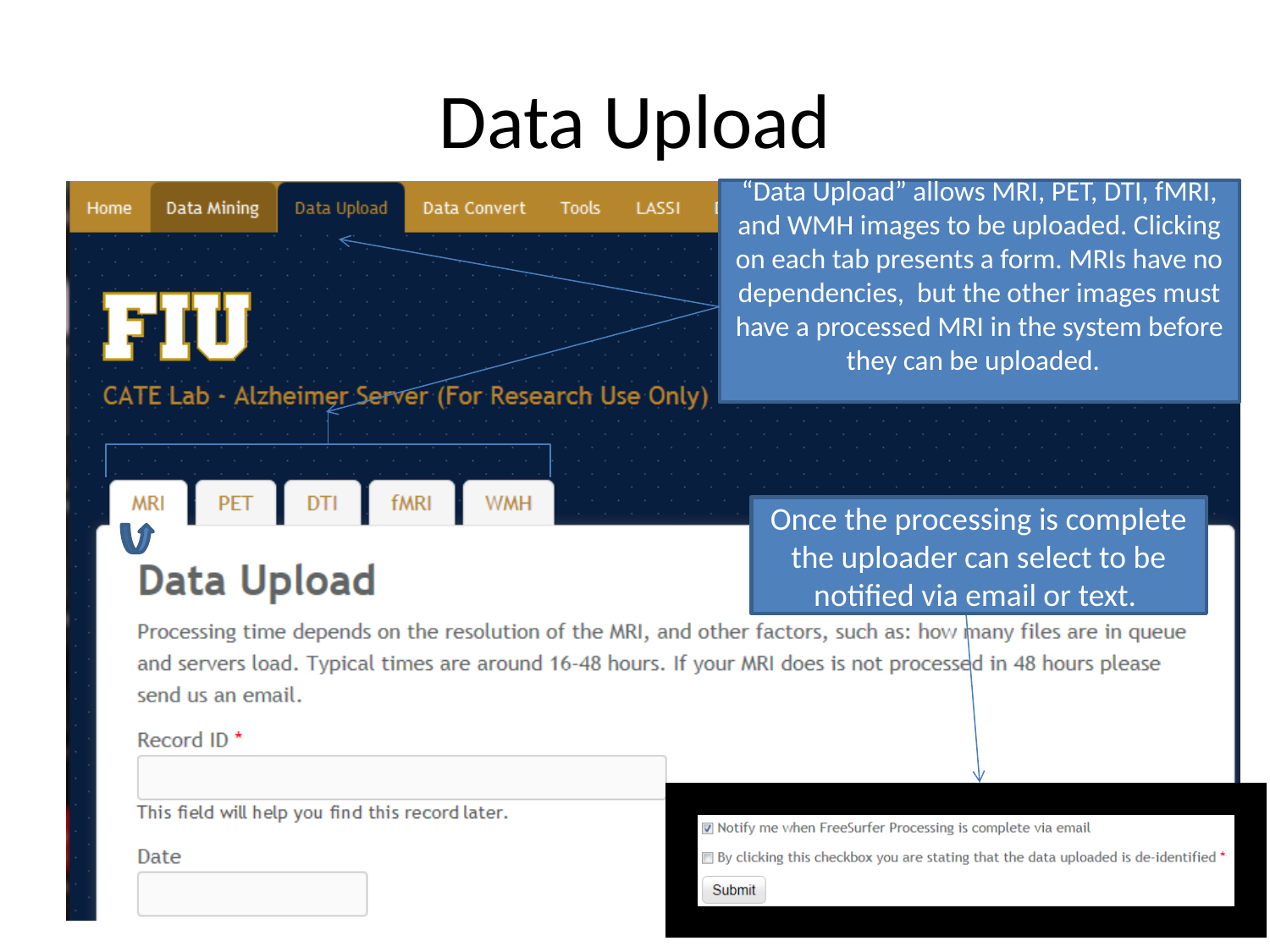

# Data Upload
“Data Upload” allows MRI, PET, DTI, fMRI, and WMH images to be uploaded. Clicking on each tab presents a form. MRIs have no dependencies, but the other images must have a processed MRI in the system before they can be uploaded.
Once the processing is complete the uploader can select to be notified via email or text.

## Slide 5
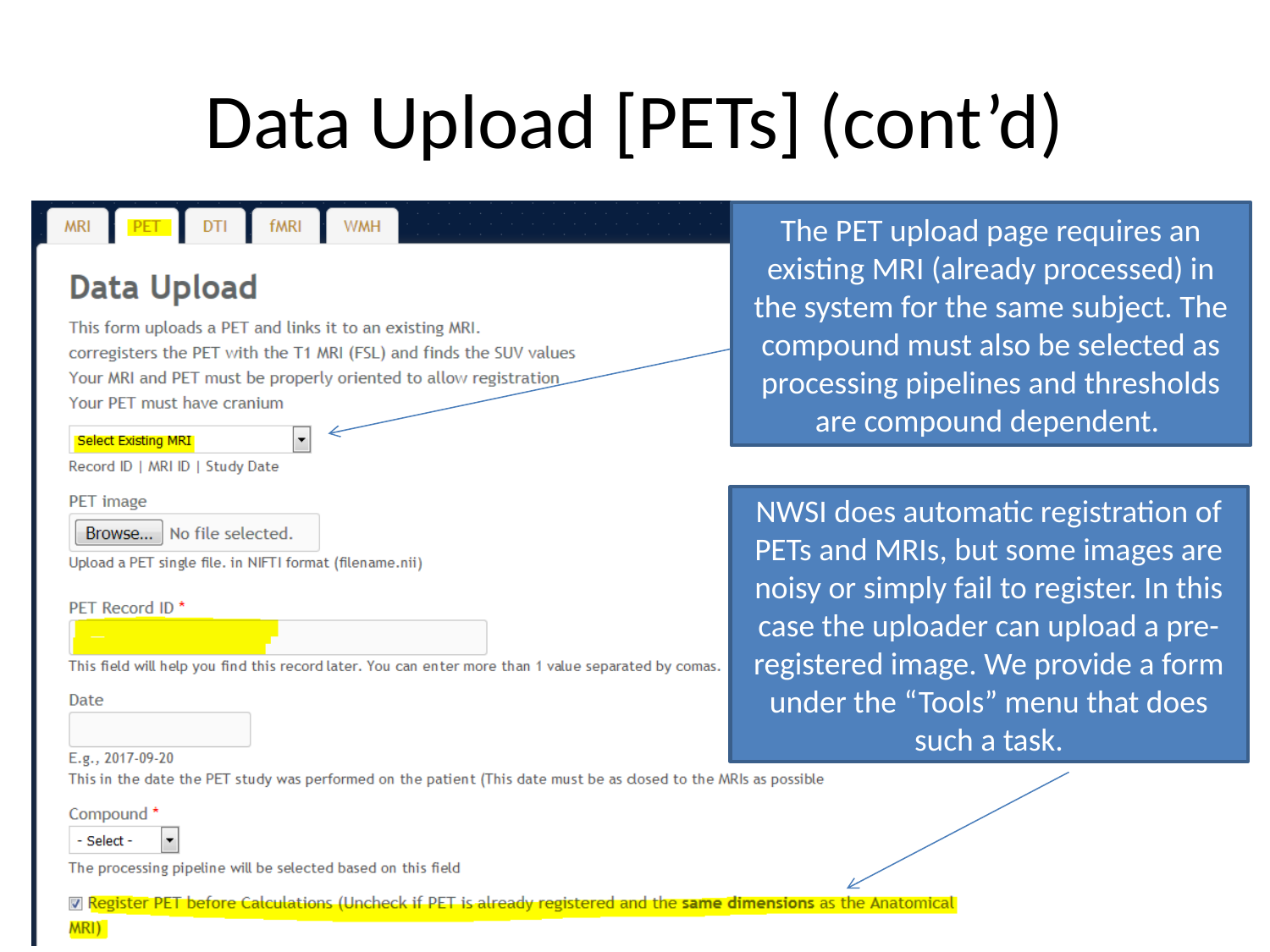

# Data Upload [PETs] (cont’d)
The PET upload page requires an existing MRI (already processed) in the system for the same subject. The compound must also be selected as processing pipelines and thresholds are compound dependent.
NWSI does automatic registration of PETs and MRIs, but some images are noisy or simply fail to register. In this case the uploader can upload a pre-registered image. We provide a form under the “Tools” menu that does such a task.

## Slide 6
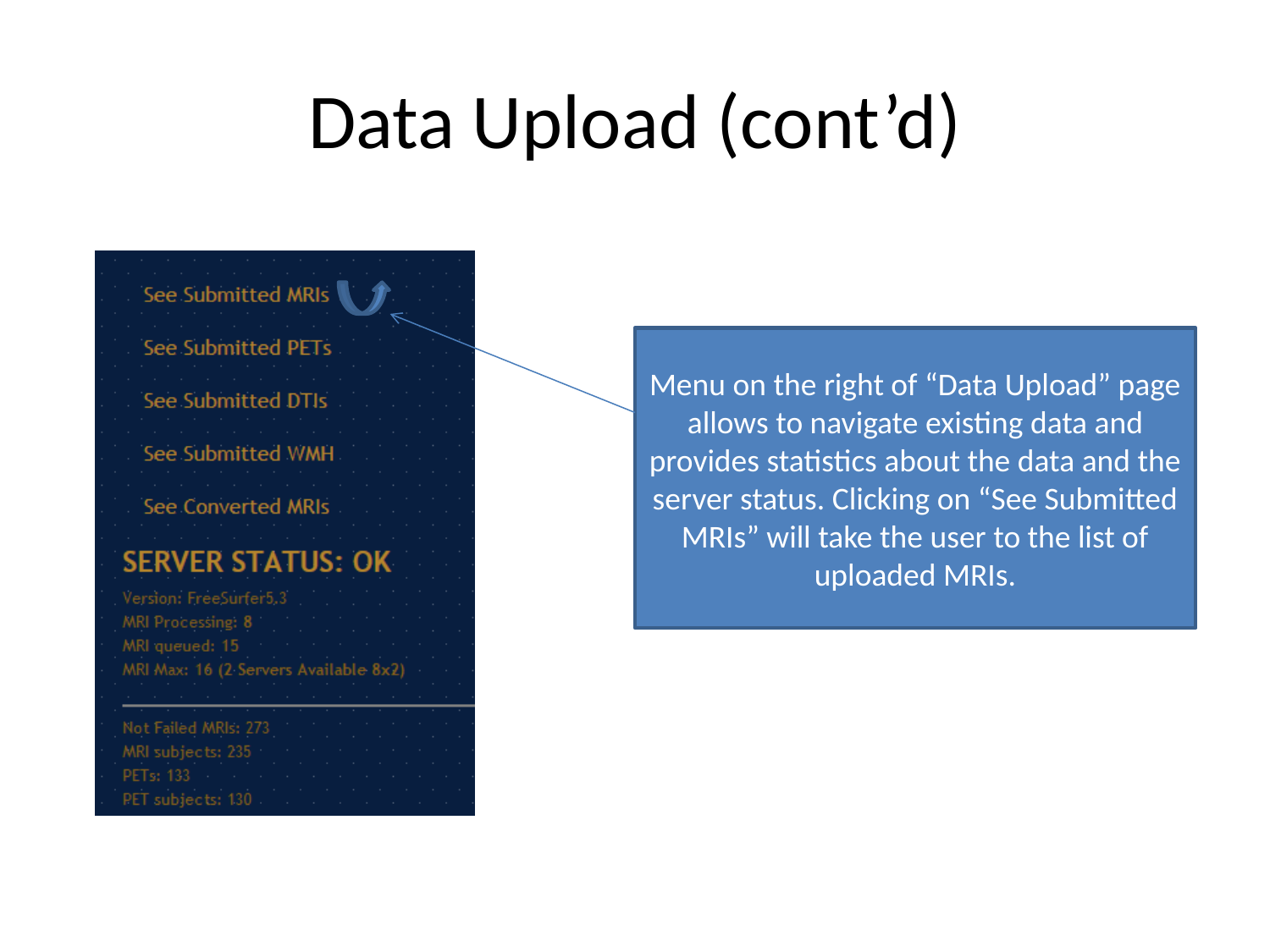

# Data Upload (cont’d)
Menu on the right of “Data Upload” page allows to navigate existing data and provides statistics about the data and the server status. Clicking on “See Submitted MRIs” will take the user to the list of uploaded MRIs.

## Slide 7
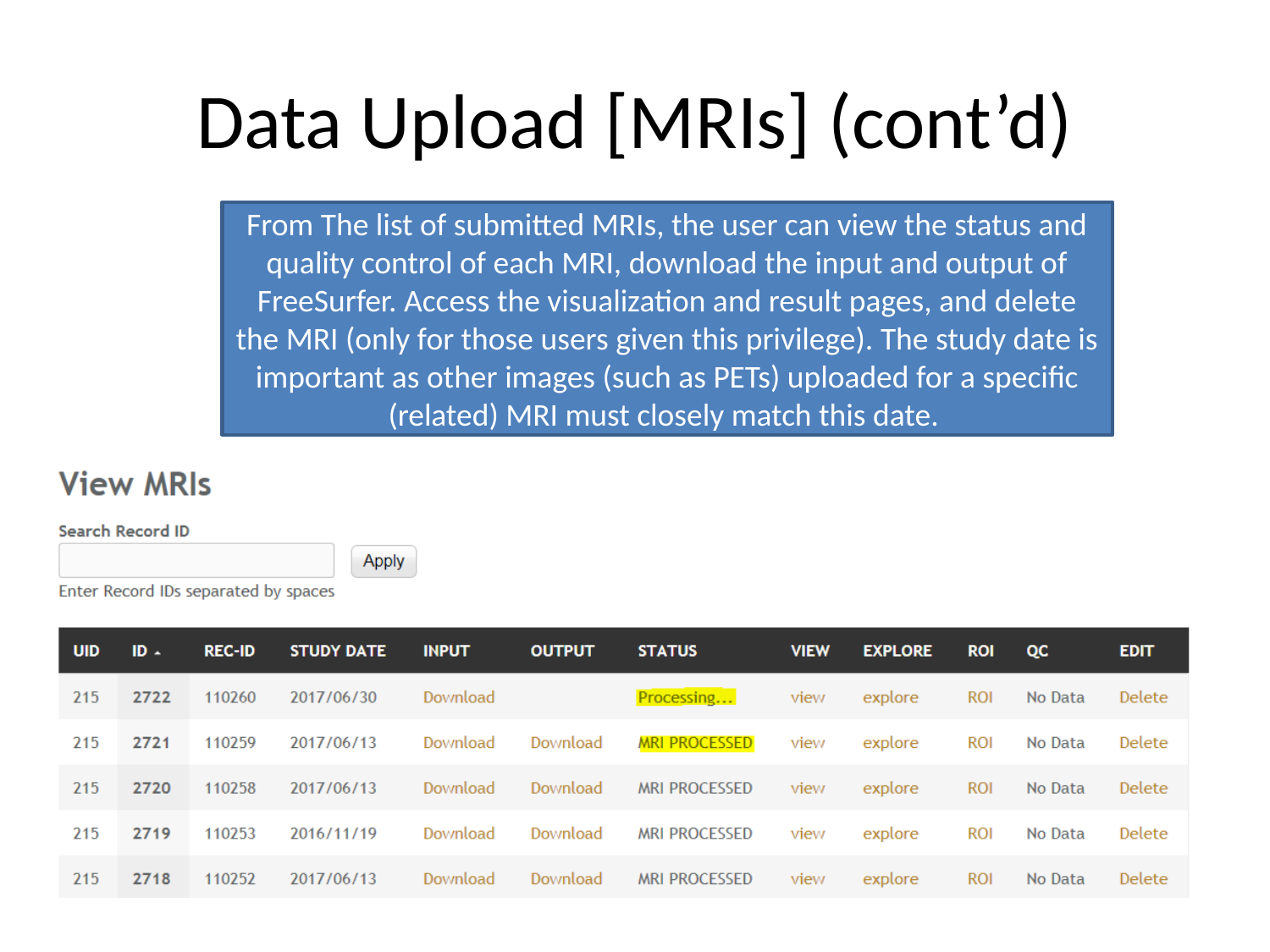

# Data Upload [MRIs] (cont’d)
From The list of submitted MRIs, the user can view the status and quality control of each MRI, download the input and output of FreeSurfer. Access the visualization and result pages, and delete the MRI (only for those users given this privilege). The study date is important as other images (such as PETs) uploaded for a specific (related) MRI must closely match this date.

## Slide 8
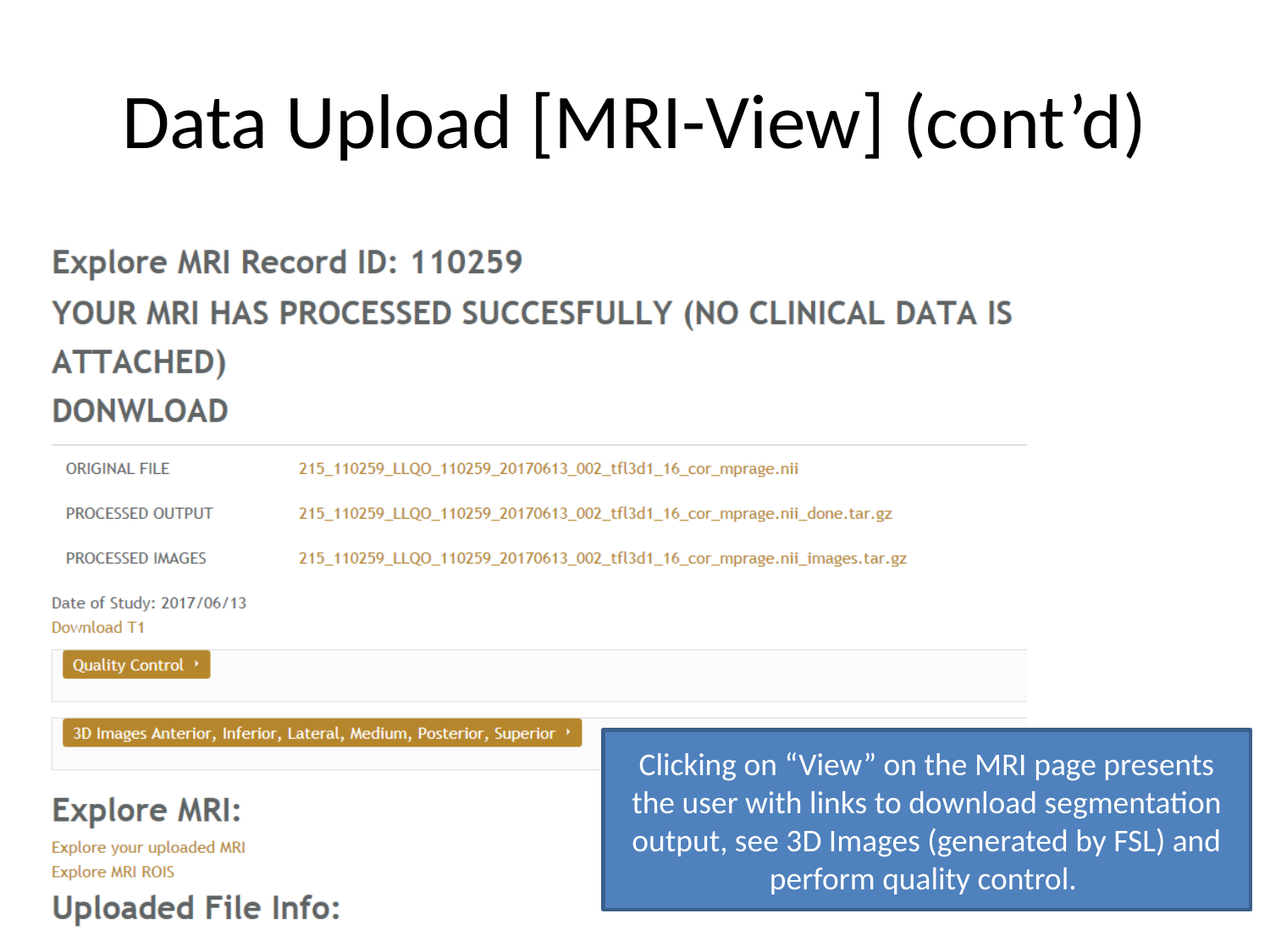

# Data Upload [MRI-View] (cont’d)
Clicking on “View” on the MRI page presents the user with links to download segmentation output, see 3D Images (generated by FSL) and perform quality control.

## Slide 9
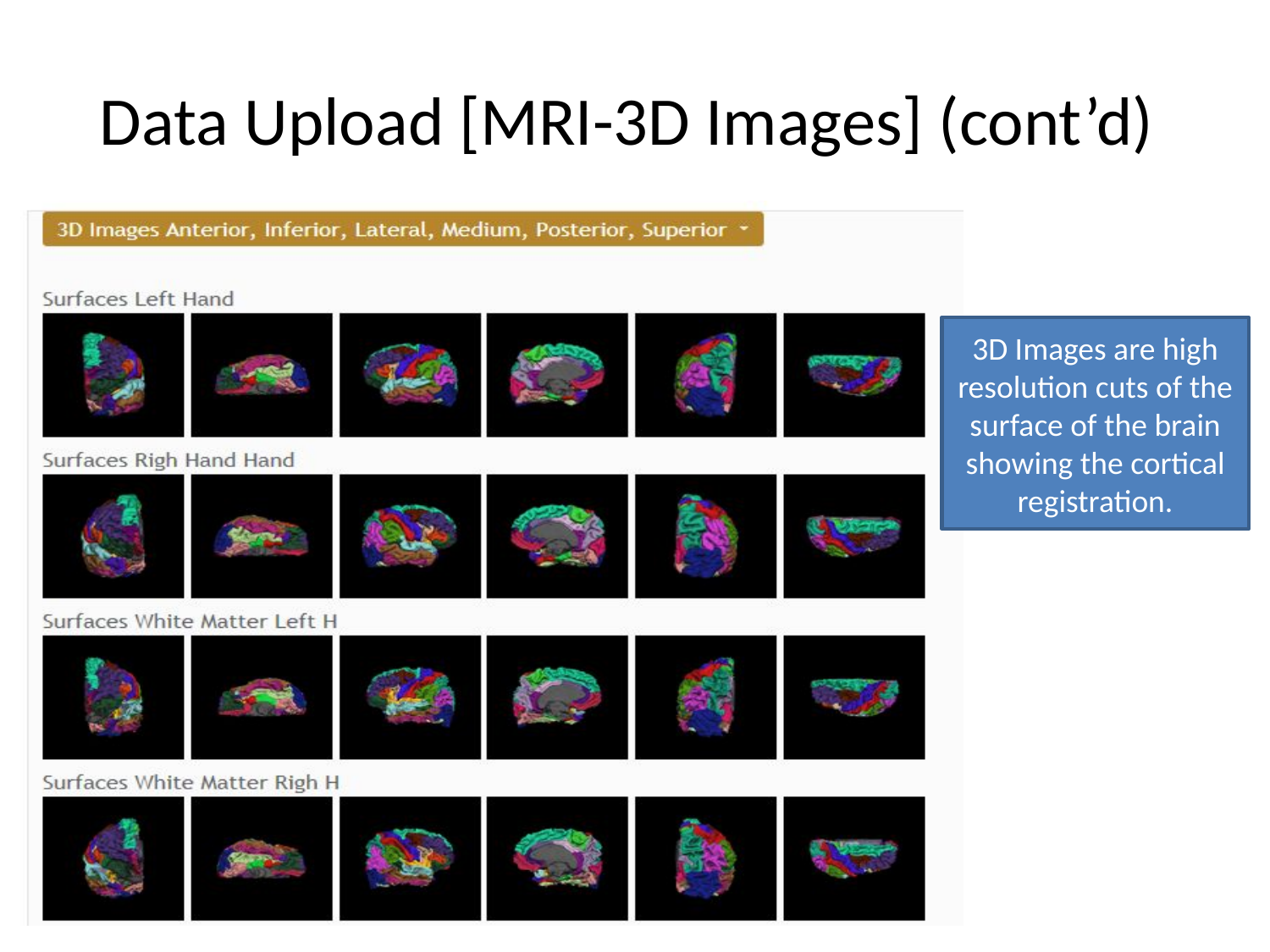

# Data Upload [MRI-3D Images] (cont’d)
3D Images are high resolution cuts of the surface of the brain showing the cortical registration.

## Slide 10
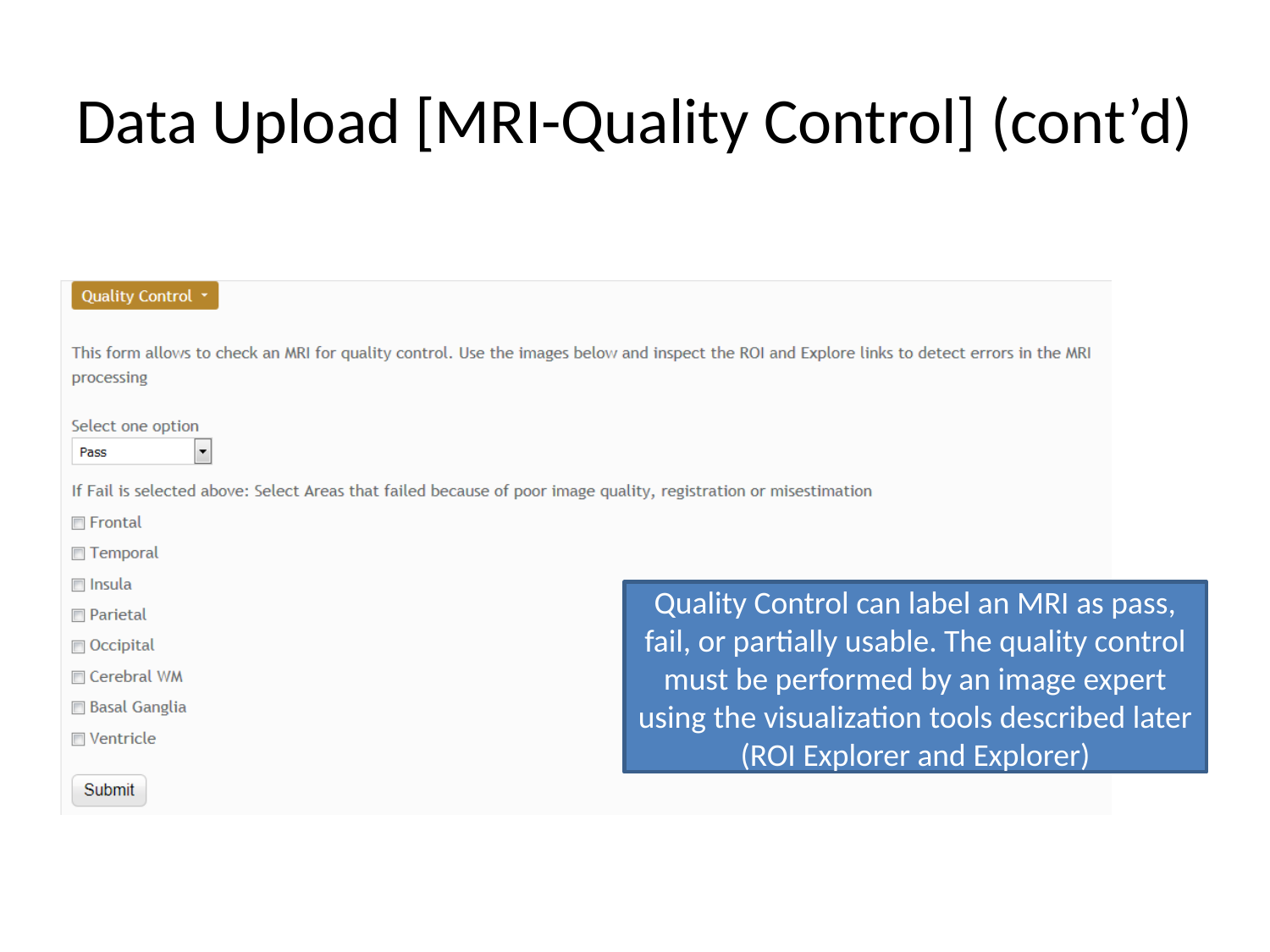

# Data Upload [MRI-Quality Control] (cont’d)
Quality Control can label an MRI as pass, fail, or partially usable. The quality control must be performed by an image expert using the visualization tools described later (ROI Explorer and Explorer)

## Slide 11
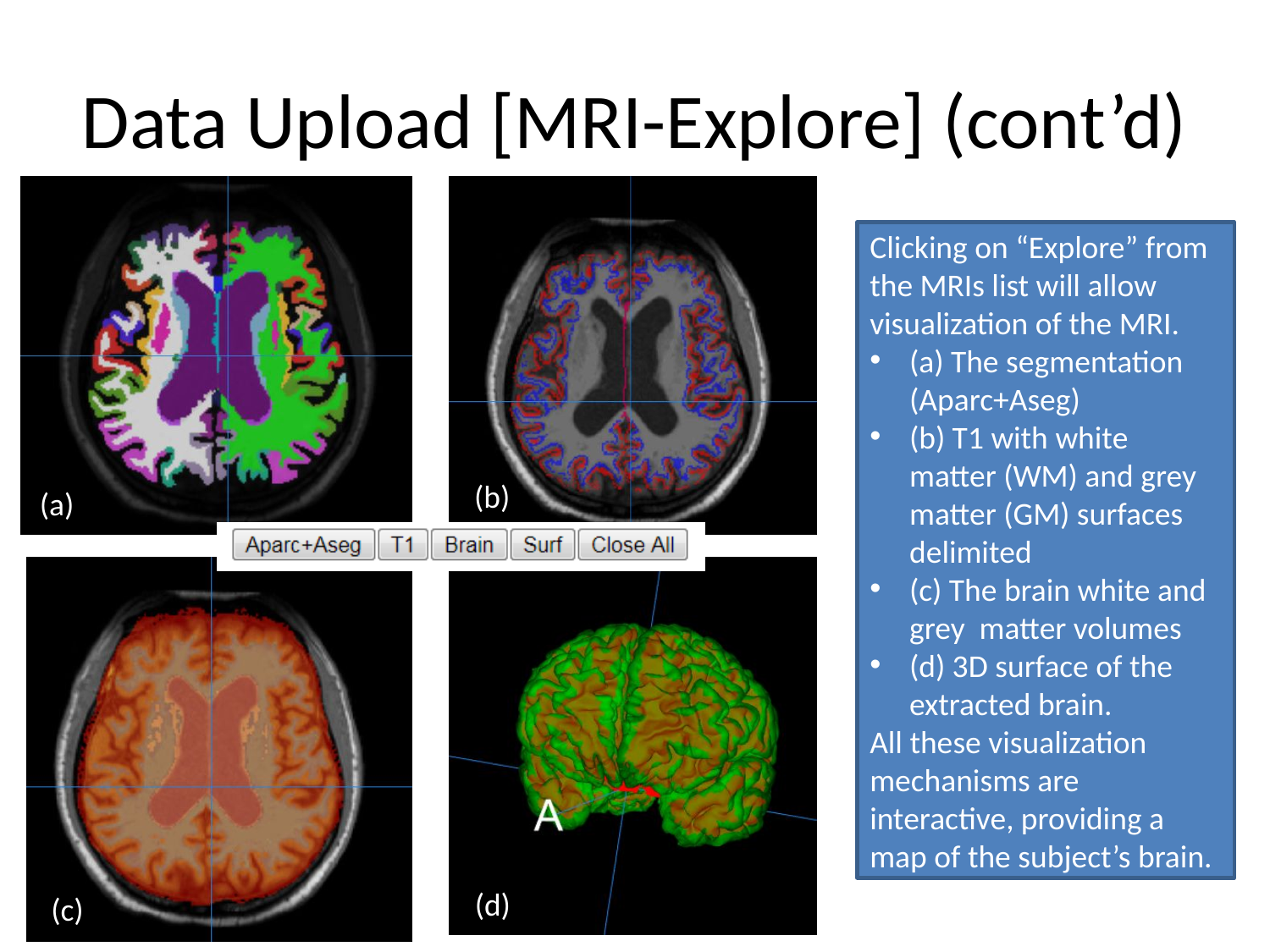

# Data Upload [MRI-Explore] (cont’d)
Clicking on “Explore” from the MRIs list will allow visualization of the MRI.
(a) The segmentation (Aparc+Aseg)
(b) T1 with white matter (WM) and grey matter (GM) surfaces delimited
(c) The brain white and grey matter volumes
(d) 3D surface of the extracted brain.
All these visualization mechanisms are interactive, providing a map of the subject’s brain.
(b)
(a)
(d)
(c)

## Slide 12
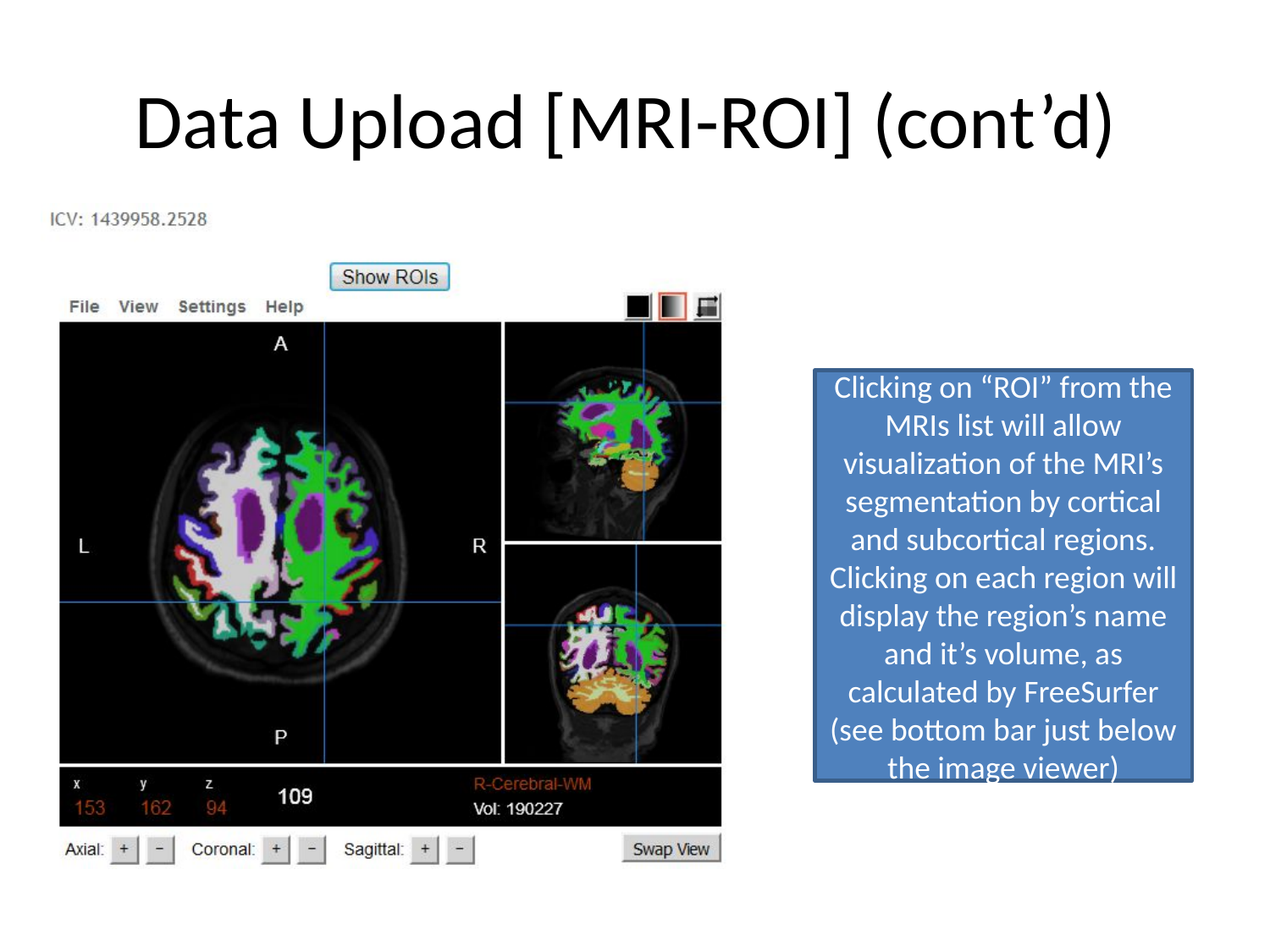

# Data Upload [MRI-ROI] (cont’d)
Clicking on “ROI” from the MRIs list will allow visualization of the MRI’s segmentation by cortical and subcortical regions. Clicking on each region will display the region’s name and it’s volume, as calculated by FreeSurfer (see bottom bar just below the image viewer)

## Slide 13
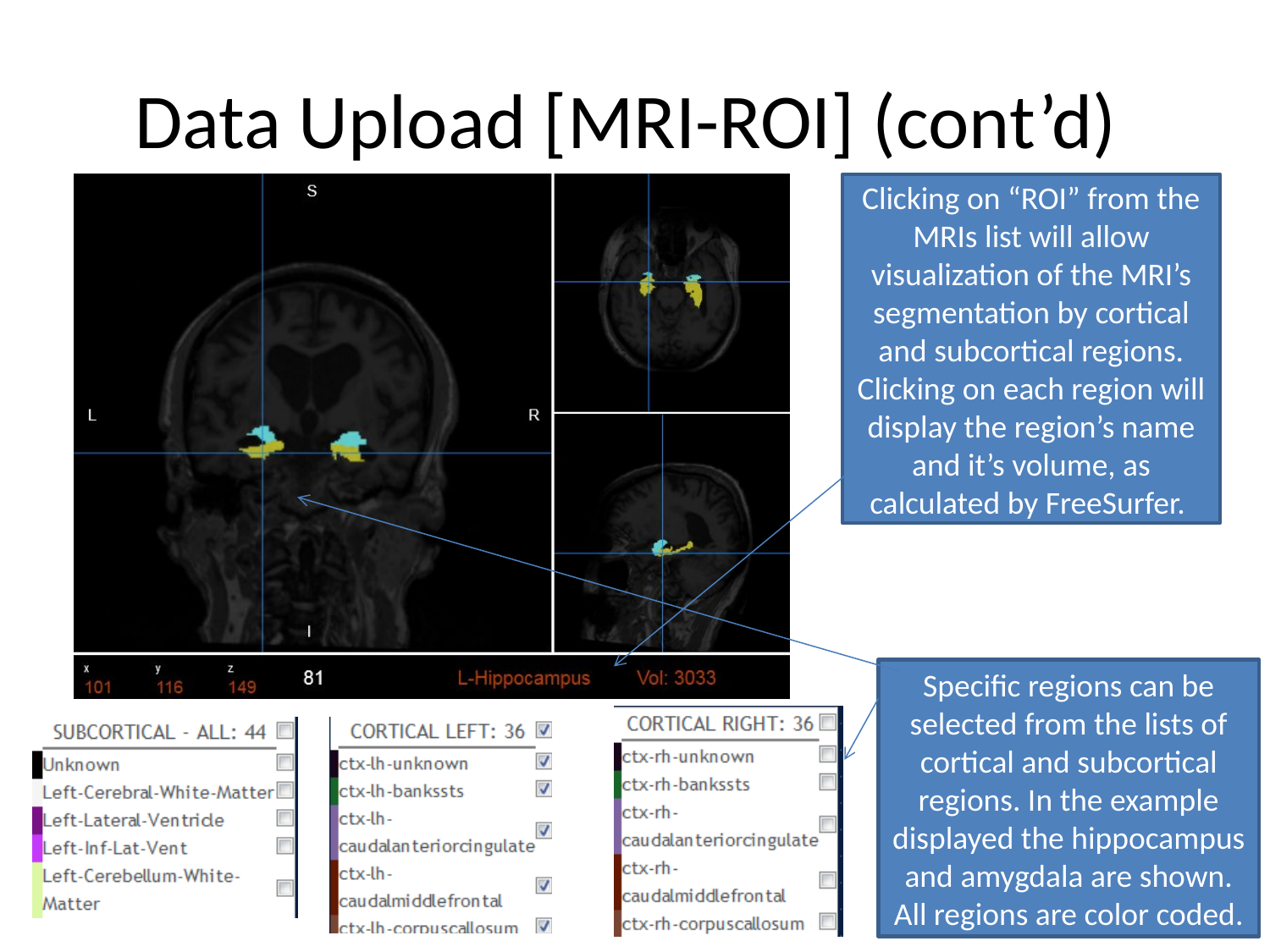

# Data Upload [MRI-ROI] (cont’d)
Clicking on “ROI” from the MRIs list will allow visualization of the MRI’s segmentation by cortical and subcortical regions. Clicking on each region will display the region’s name and it’s volume, as calculated by FreeSurfer.
Specific regions can be selected from the lists of cortical and subcortical regions. In the example displayed the hippocampus and amygdala are shown. All regions are color coded.

## Slide 14
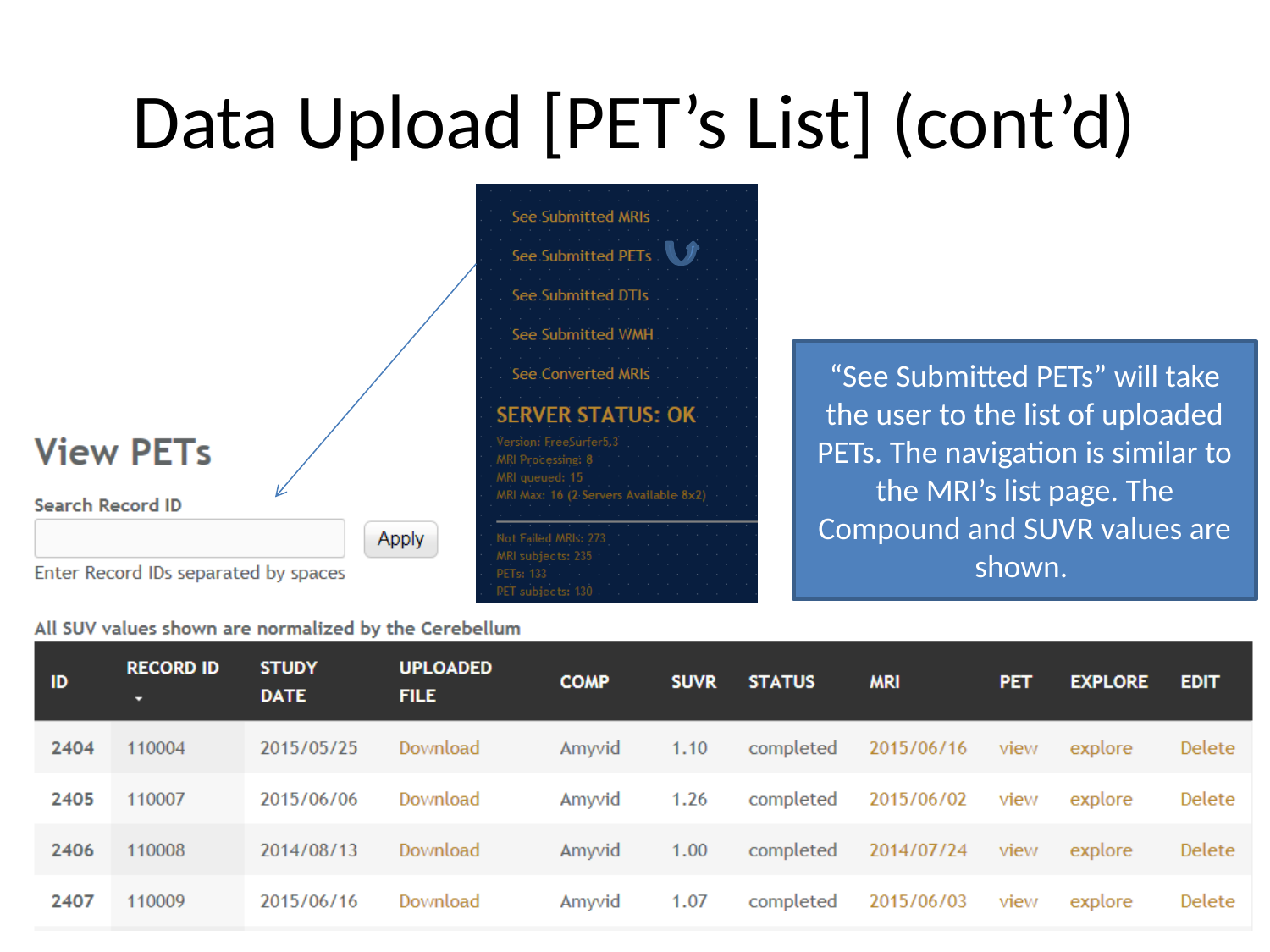

# Data Upload [PET’s List] (cont’d)
“See Submitted PETs” will take the user to the list of uploaded PETs. The navigation is similar to the MRI’s list page. The Compound and SUVR values are shown.

## Slide 15
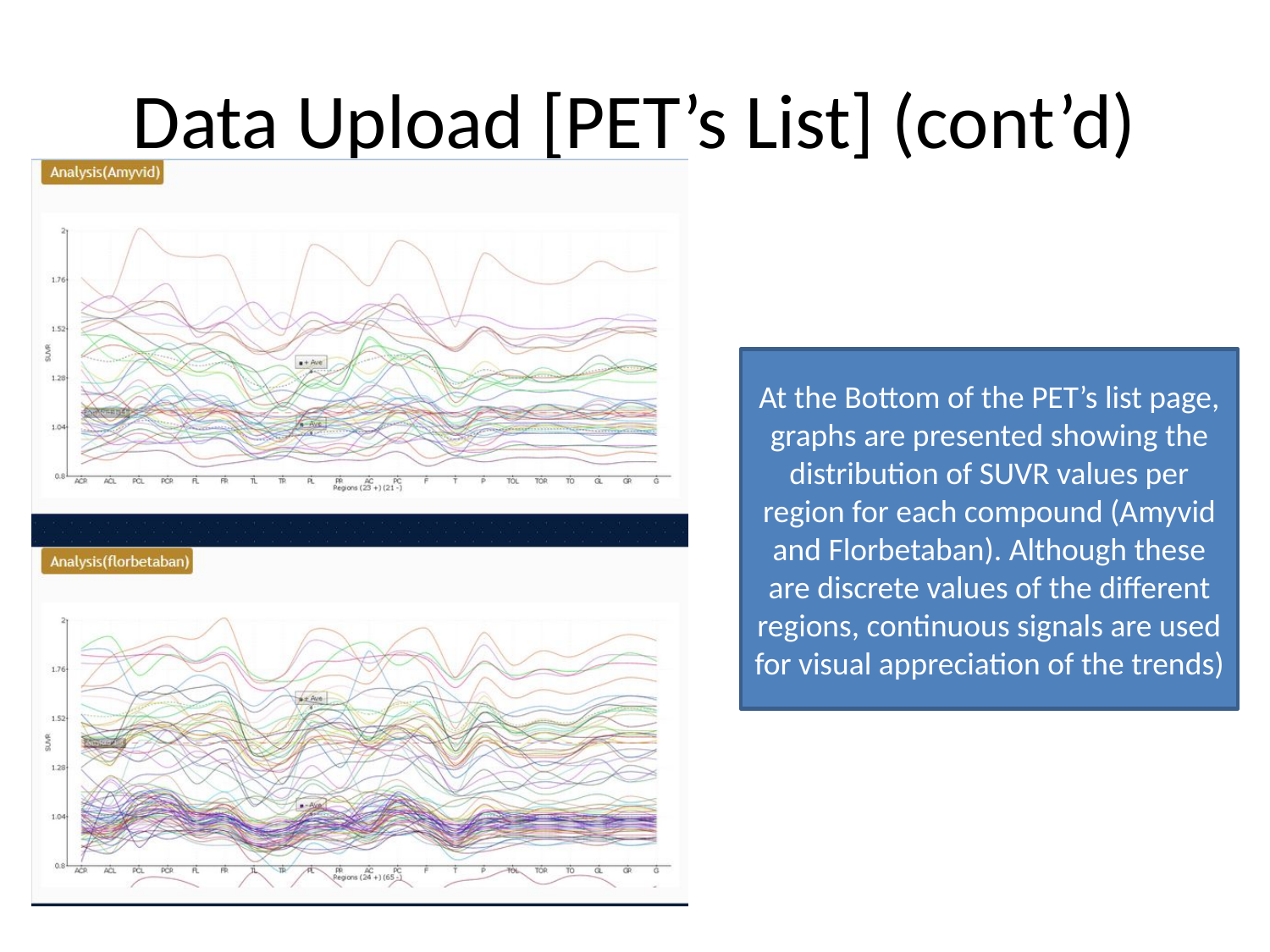

# Data Upload [PET’s List] (cont’d)
At the Bottom of the PET’s list page, graphs are presented showing the distribution of SUVR values per region for each compound (Amyvid and Florbetaban). Although these are discrete values of the different regions, continuous signals are used for visual appreciation of the trends)

## Slide 16
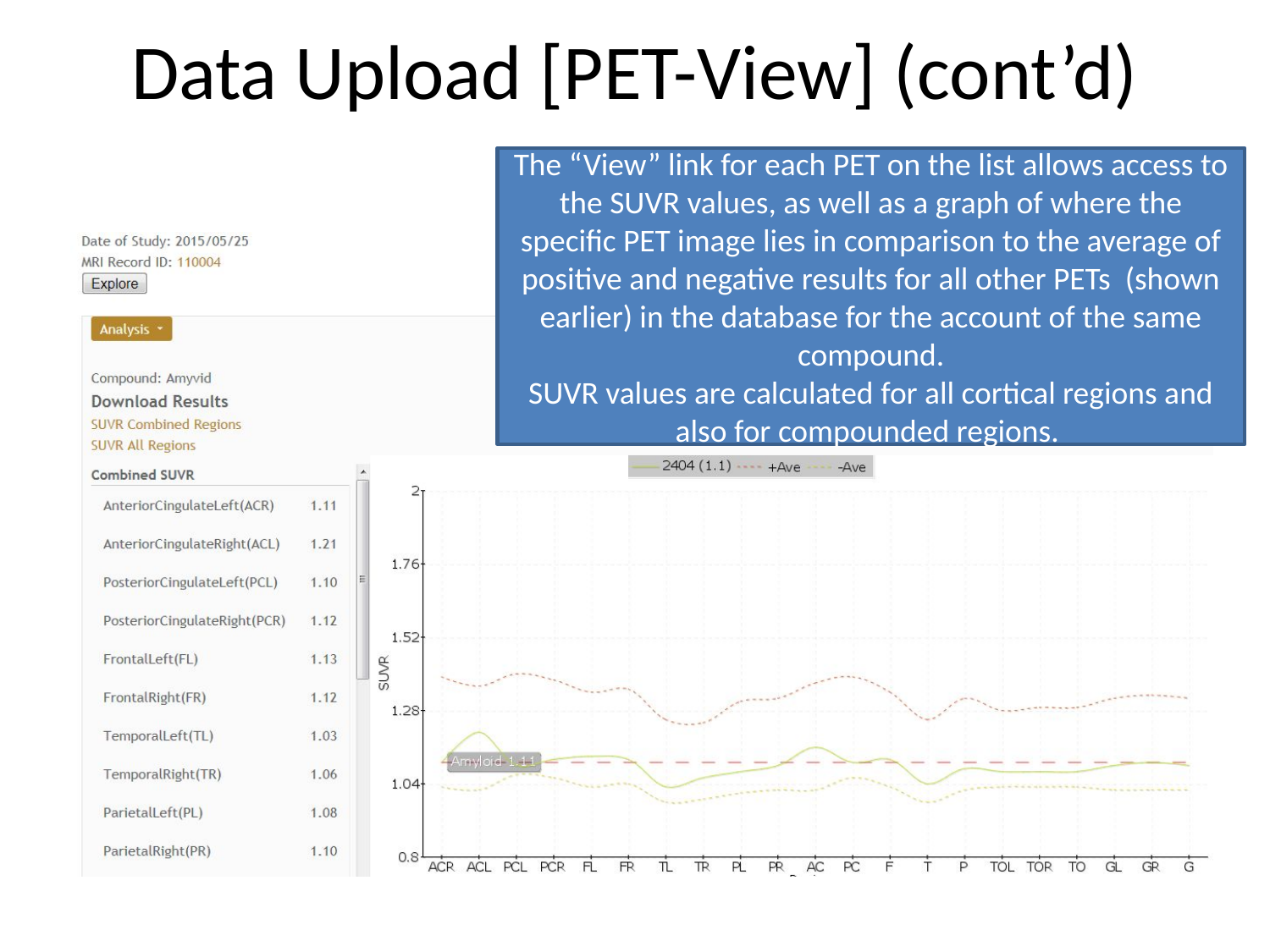

# Data Upload [PET-View] (cont’d)
The “View” link for each PET on the list allows access to the SUVR values, as well as a graph of where the specific PET image lies in comparison to the average of positive and negative results for all other PETs (shown earlier) in the database for the account of the same compound.
SUVR values are calculated for all cortical regions and also for compounded regions.

## Slide 17
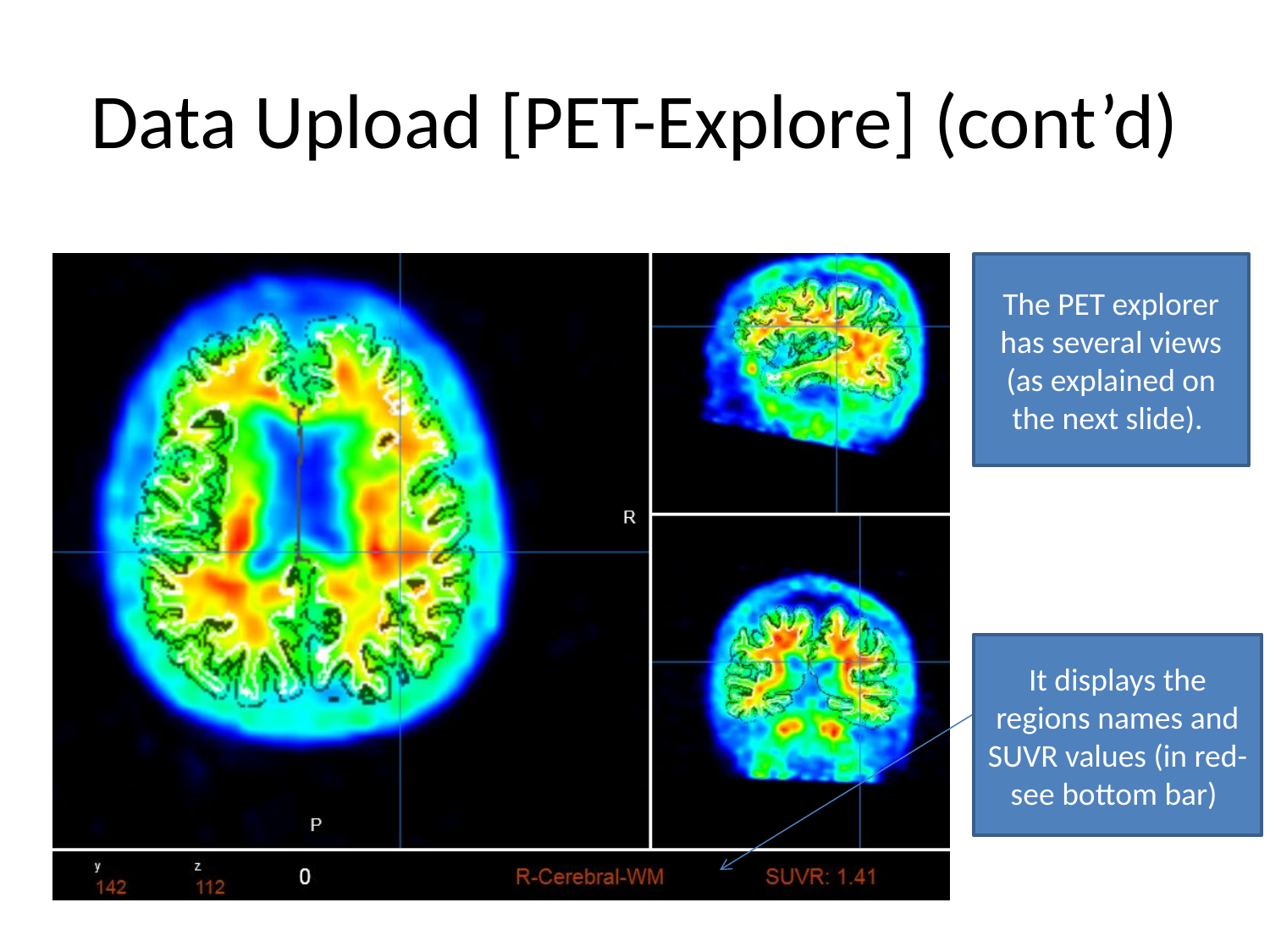

# Data Upload [PET-Explore] (cont’d)
The PET explorer has several views (as explained on the next slide).
It displays the regions names and SUVR values (in red-see bottom bar)

## Slide 18
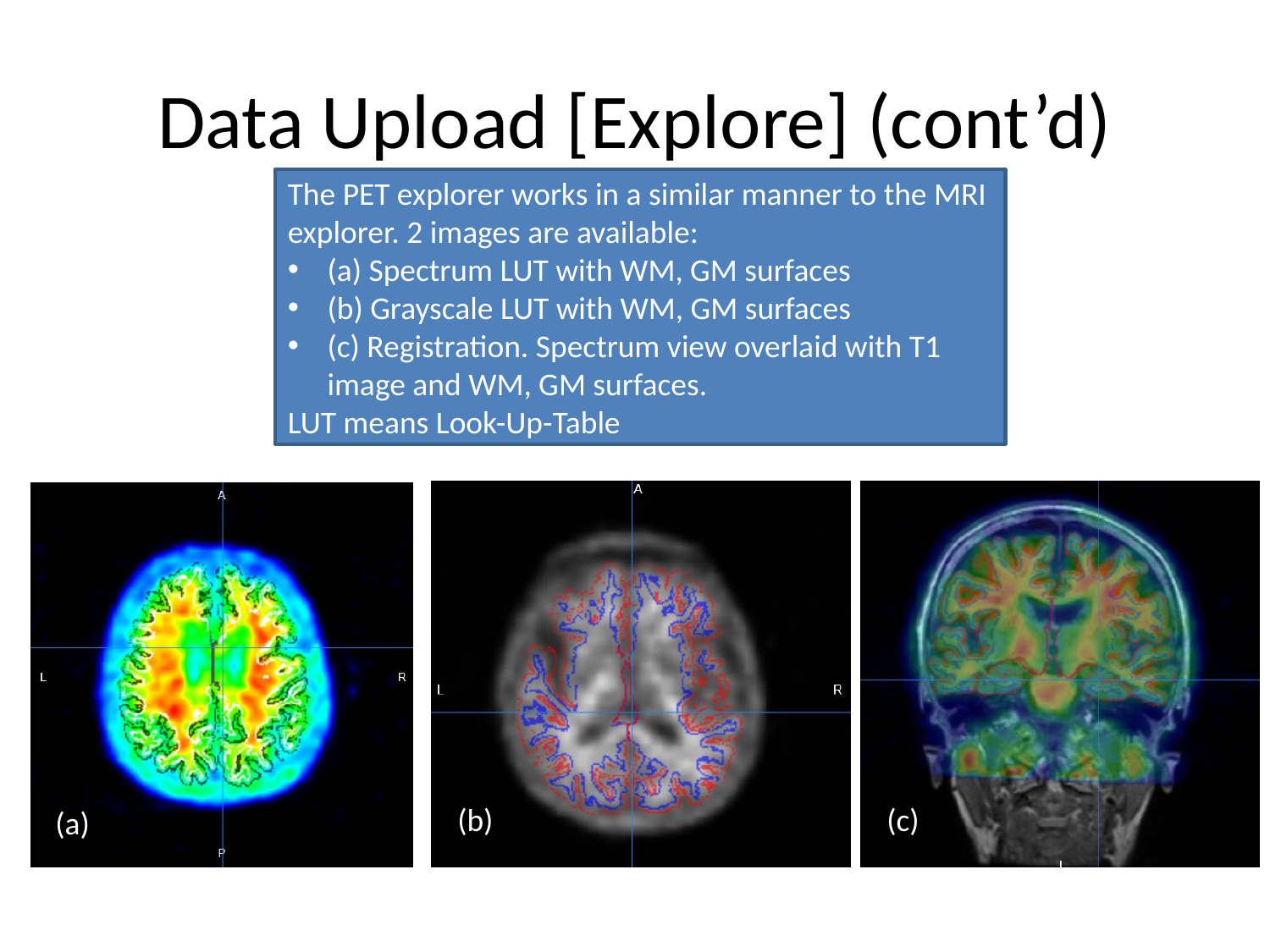

# Data Upload [Explore] (cont’d)
The PET explorer works in a similar manner to the MRI explorer. 2 images are available:
(a) Spectrum LUT with WM, GM surfaces
(b) Grayscale LUT with WM, GM surfaces
(c) Registration. Spectrum view overlaid with T1 image and WM, GM surfaces.
LUT means Look-Up-Table
(b)
(c)
(a)

## Slide 19
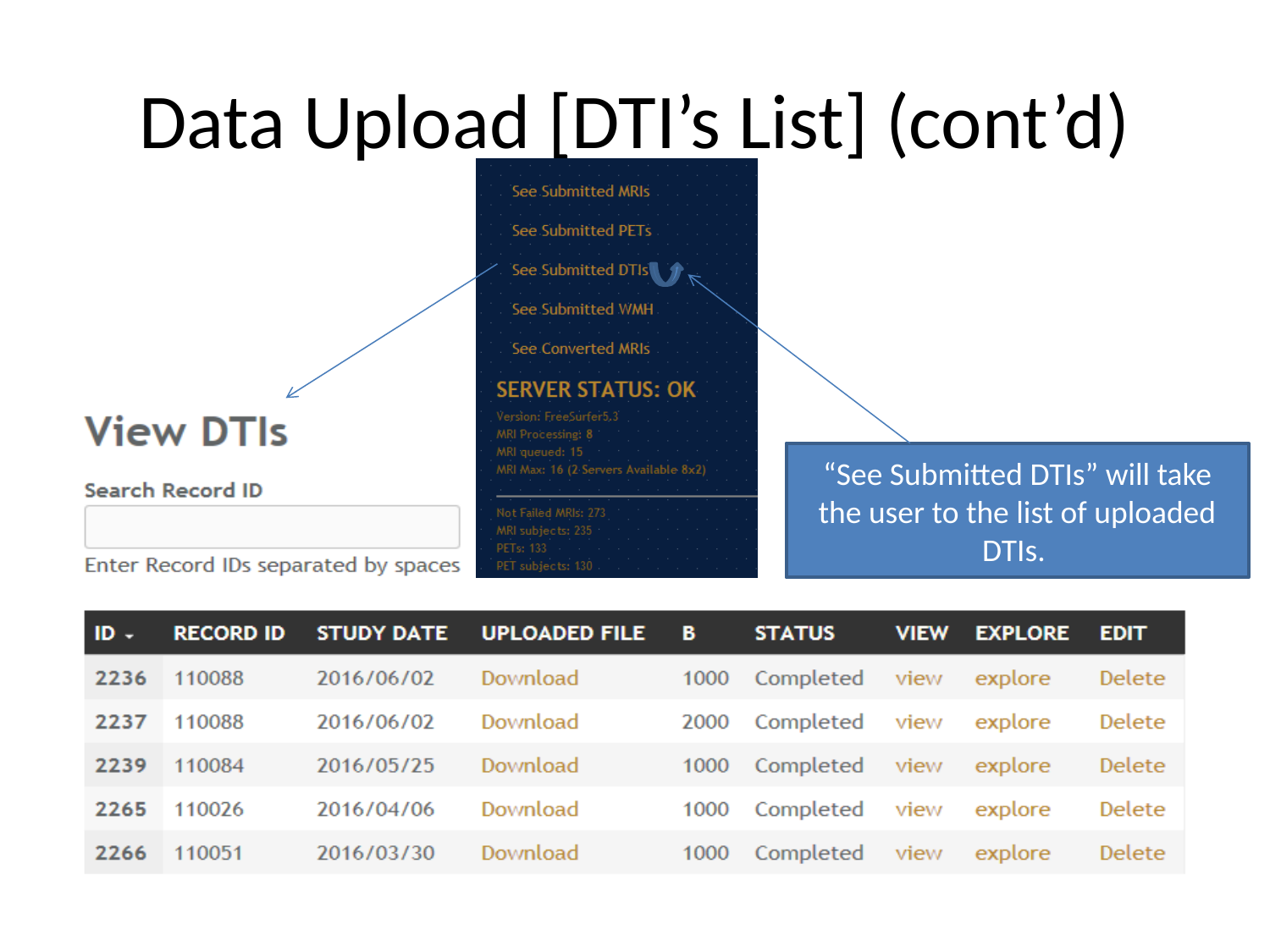

# Data Upload [DTI’s List] (cont’d)
“See Submitted DTIs” will take the user to the list of uploaded DTIs.

## Slide 20
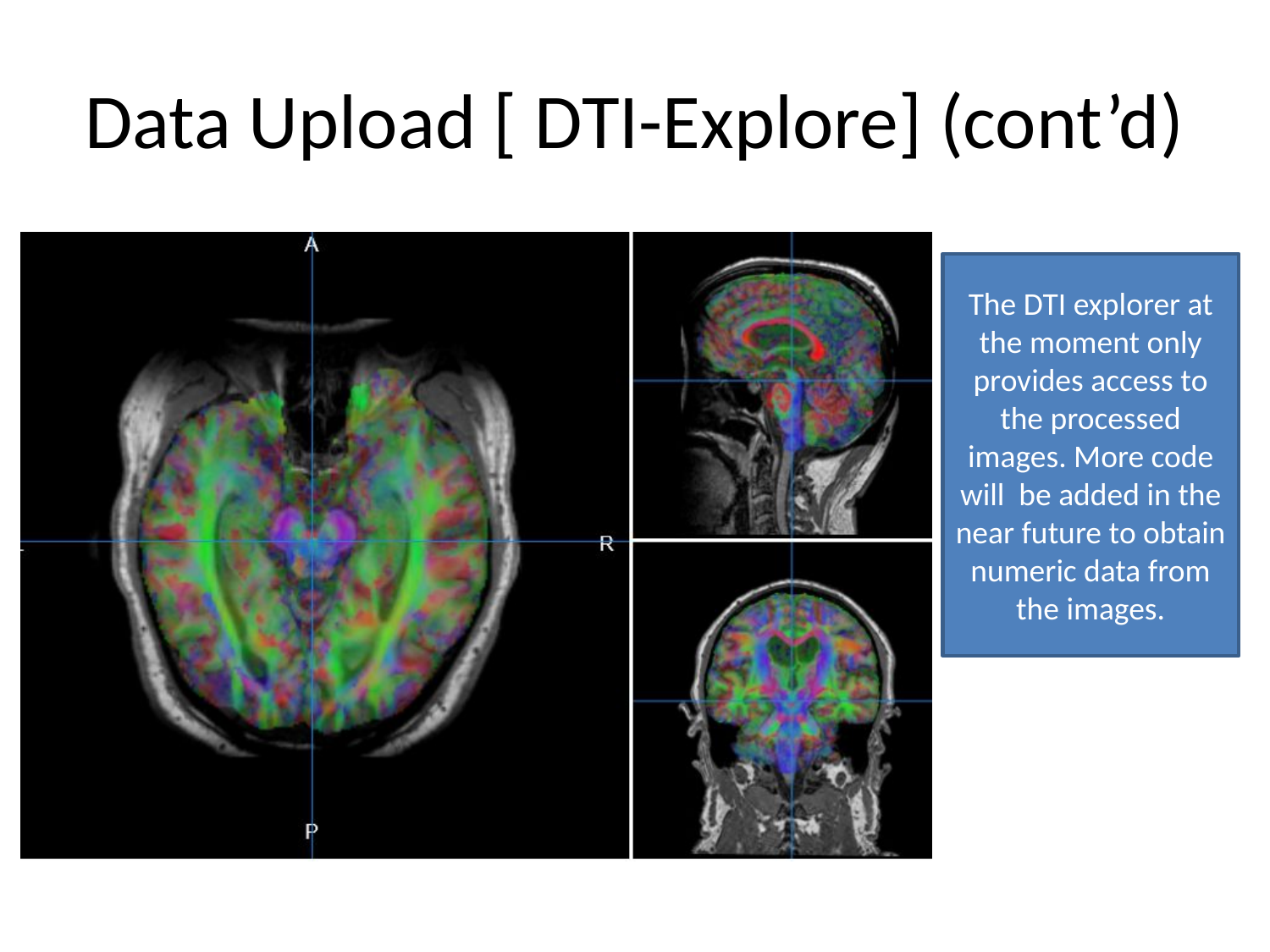

# Data Upload [ DTI-Explore] (cont’d)
The DTI explorer at the moment only provides access to the processed images. More code will be added in the near future to obtain numeric data from the images.

## Slide 21
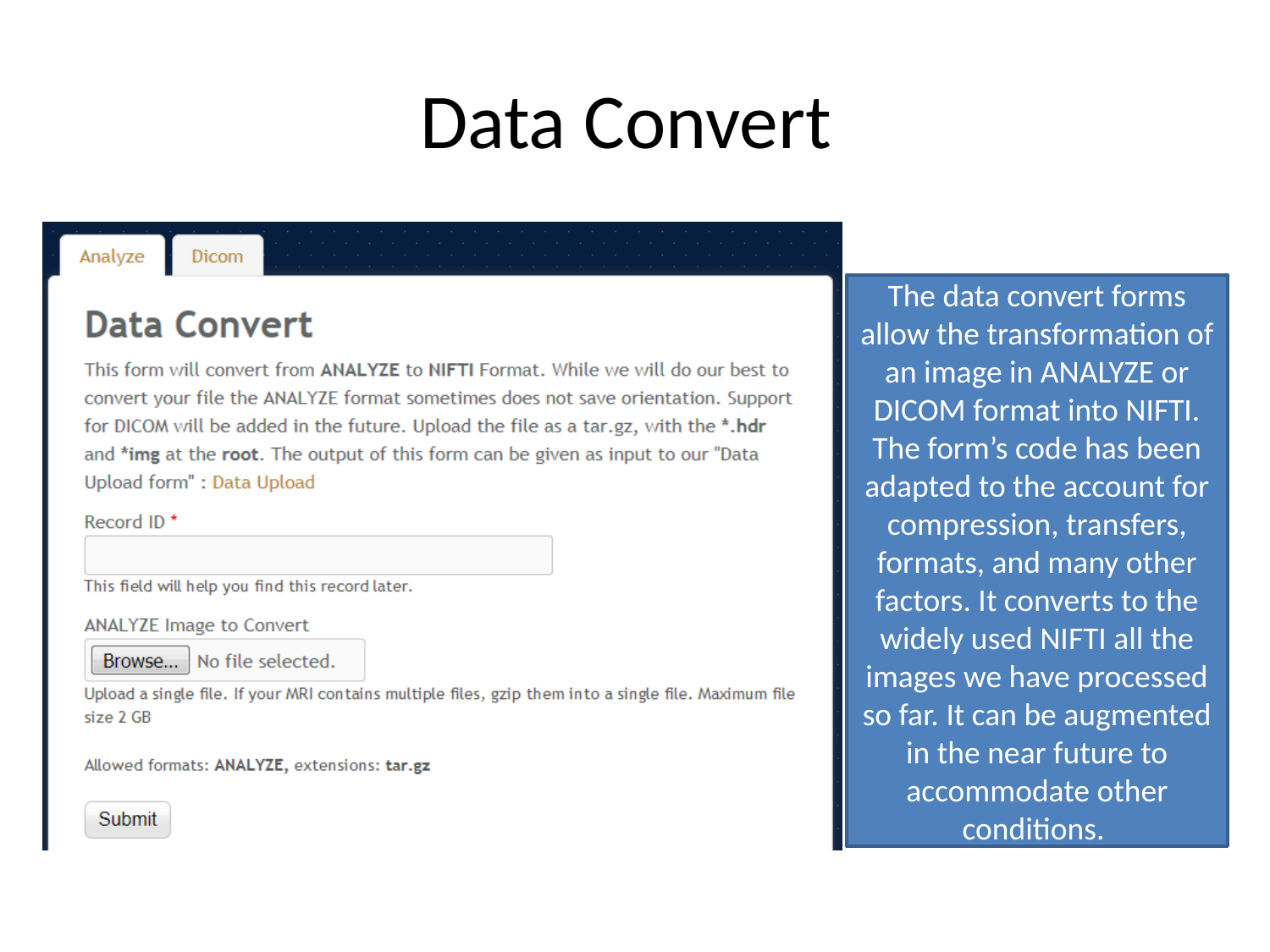

# Data Convert
The data convert forms allow the transformation of an image in ANALYZE or DICOM format into NIFTI. The form’s code has been adapted to the account for compression, transfers, formats, and many other factors. It converts to the widely used NIFTI all the images we have processed so far. It can be augmented in the near future to accommodate other conditions.

## Slide 22
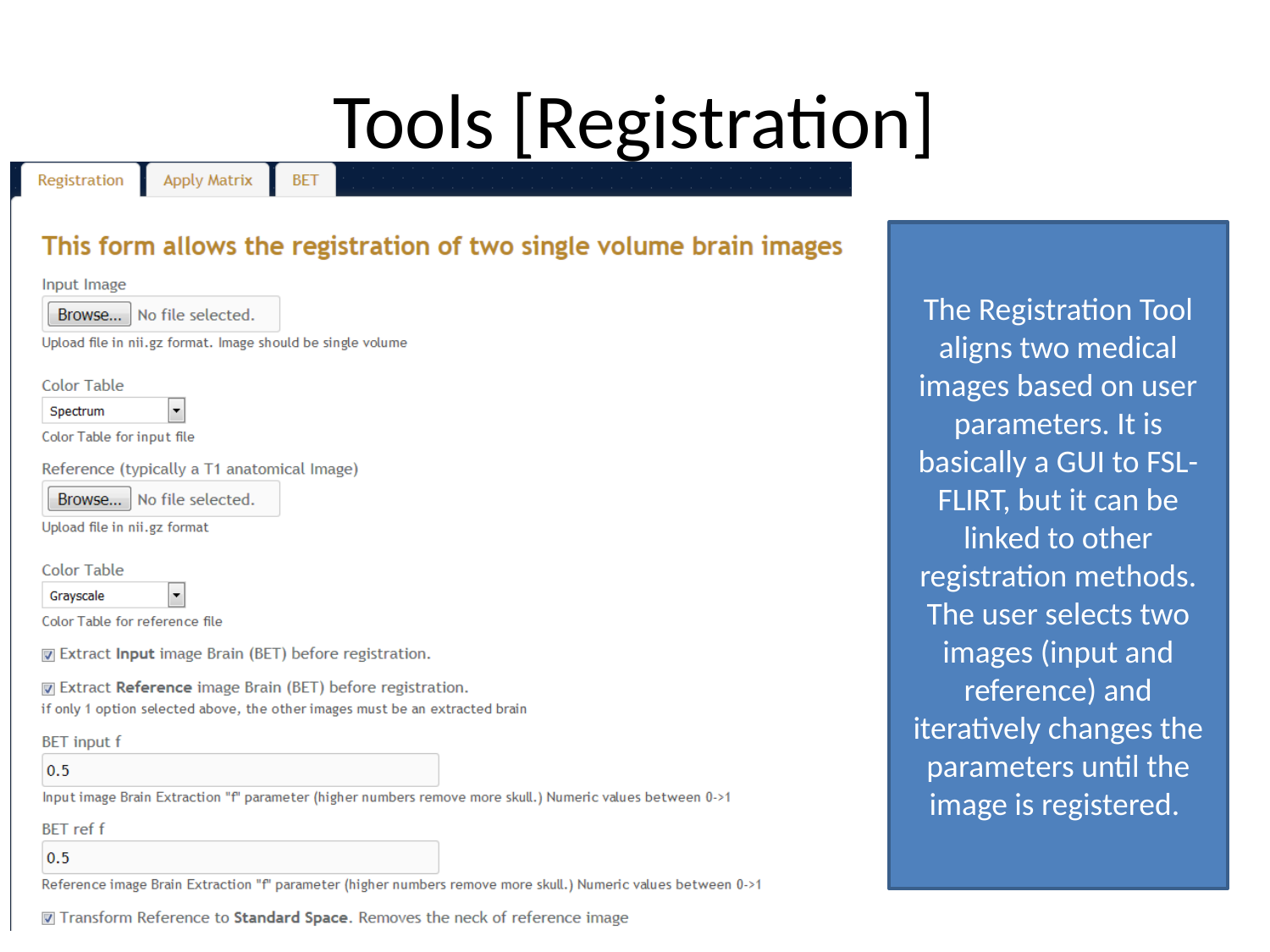

# Tools [Registration]
The Registration Tool aligns two medical images based on user parameters. It is basically a GUI to FSL-FLIRT, but it can be linked to other registration methods. The user selects two images (input and reference) and iteratively changes the parameters until the image is registered.

## Slide 23
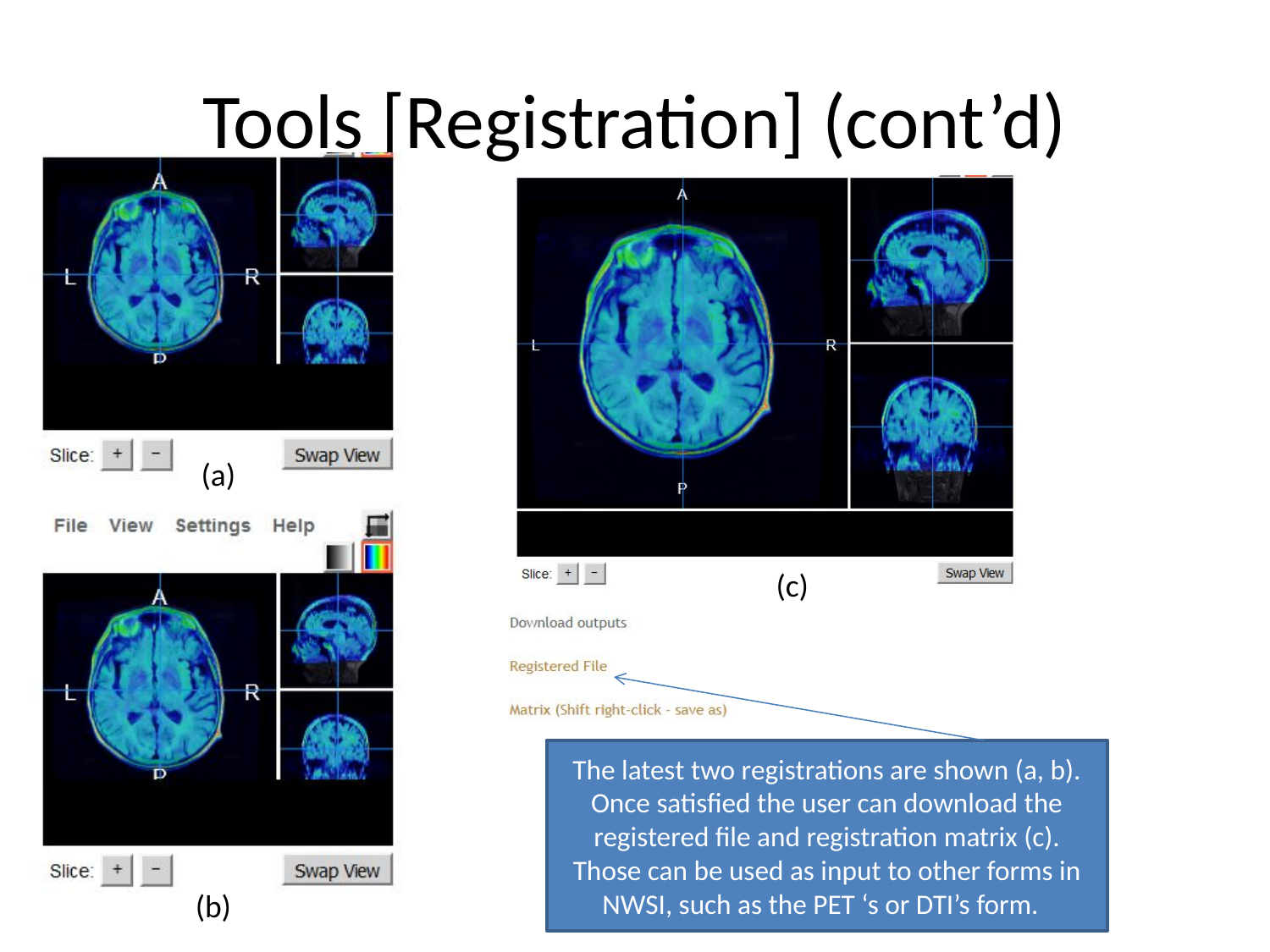

# Tools [Registration] (cont’d)
(a)
(c)
The latest two registrations are shown (a, b). Once satisfied the user can download the registered file and registration matrix (c). Those can be used as input to other forms in NWSI, such as the PET ‘s or DTI’s form.
(b)

## Slide 24
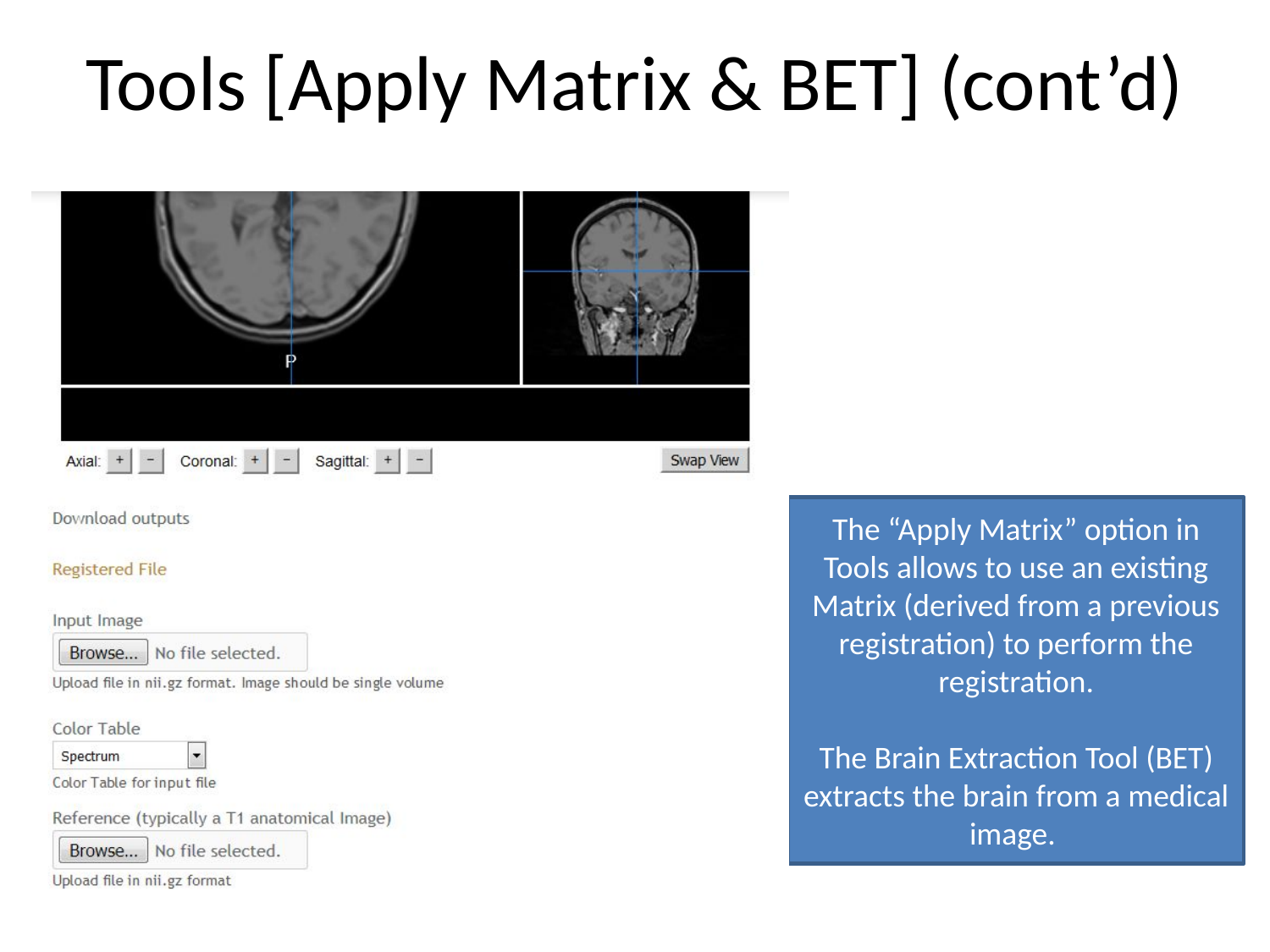

# Tools [Apply Matrix & BET] (cont’d)
The “Apply Matrix” option in Tools allows to use an existing Matrix (derived from a previous registration) to perform the registration.
The Brain Extraction Tool (BET) extracts the brain from a medical image.

## Slide 25
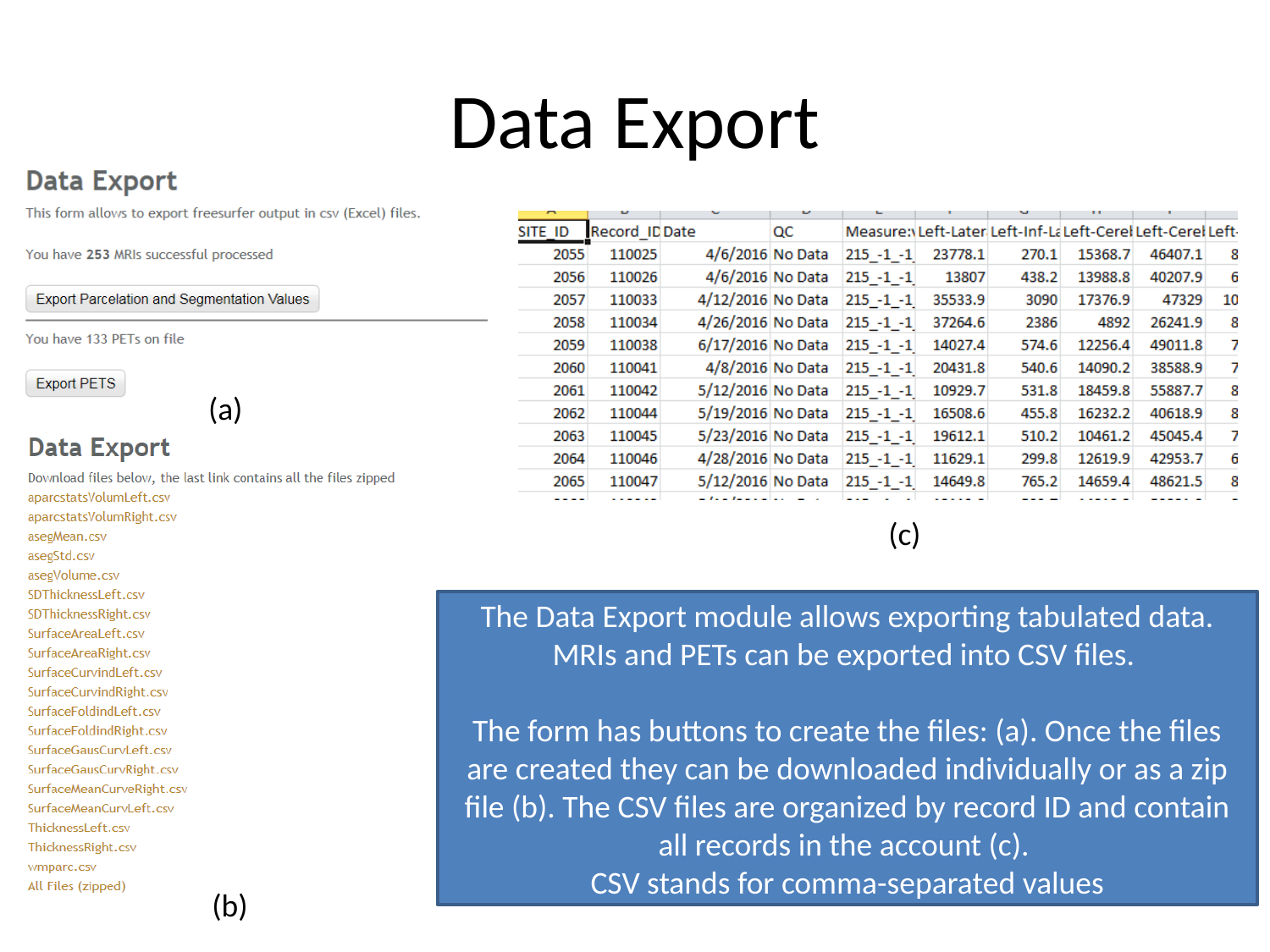

# Data Export
(a)
(c)
The Data Export module allows exporting tabulated data. MRIs and PETs can be exported into CSV files.
The form has buttons to create the files: (a). Once the files are created they can be downloaded individually or as a zip file (b). The CSV files are organized by record ID and contain all records in the account (c).
CSV stands for comma-separated values
(a)
(b)

## Slide 26
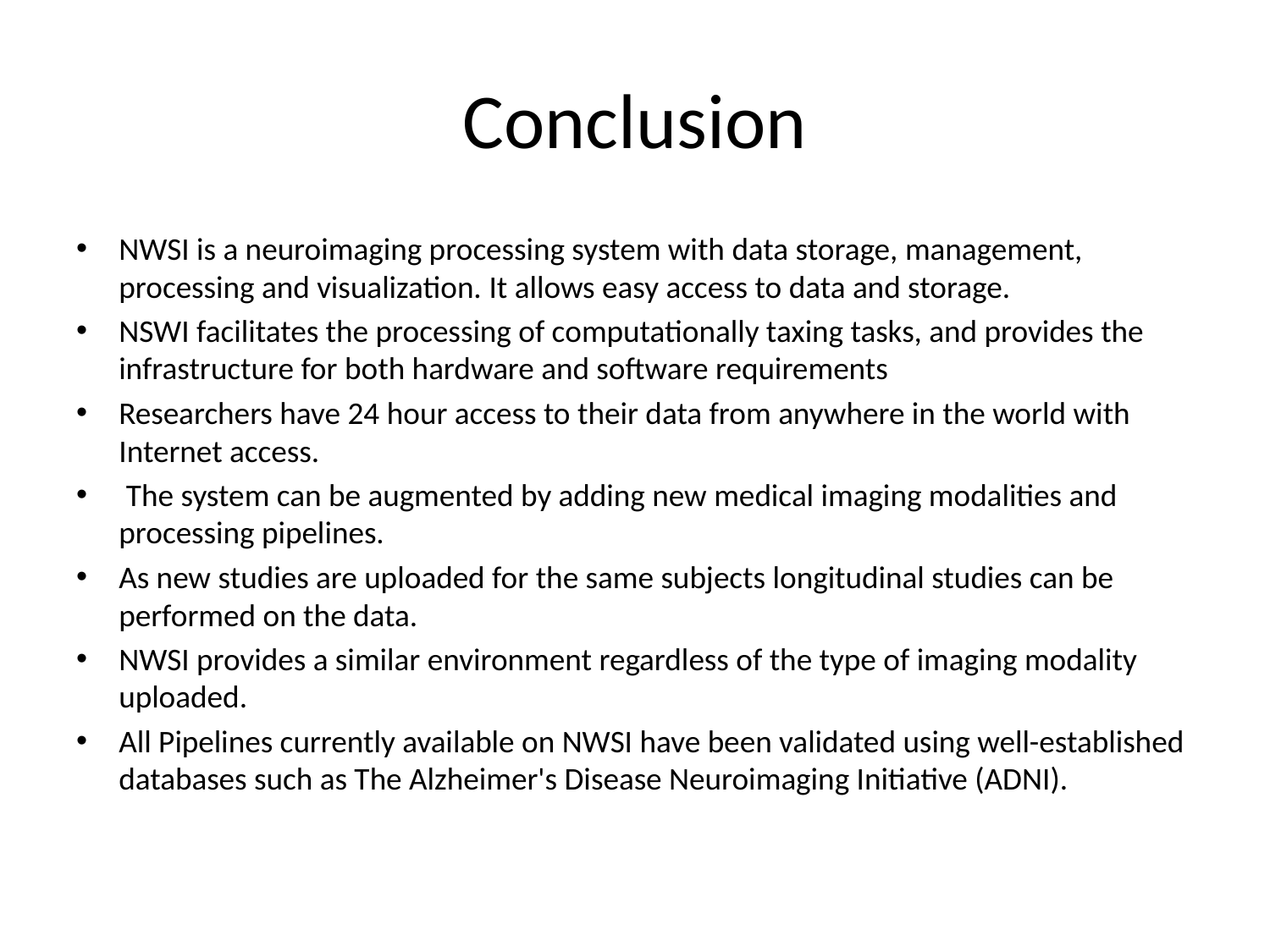

# Conclusion
NWSI is a neuroimaging processing system with data storage, management, processing and visualization. It allows easy access to data and storage.
NSWI facilitates the processing of computationally taxing tasks, and provides the infrastructure for both hardware and software requirements
Researchers have 24 hour access to their data from anywhere in the world with Internet access.
 The system can be augmented by adding new medical imaging modalities and processing pipelines.
As new studies are uploaded for the same subjects longitudinal studies can be performed on the data.
NWSI provides a similar environment regardless of the type of imaging modality uploaded.
All Pipelines currently available on NWSI have been validated using well-established databases such as The Alzheimer's Disease Neuroimaging Initiative (ADNI).
